# Association of obesity-metabolic indices with heart failure in patients with coronary artery disease: a multicenter retrospective observational study

**DOI:** 10.3389/fendo.2026.1826249

**Published:** 2026-04-17

**Authors:** Xianwei Tian, Penghui Cui, Yazhe Liu, Zhiqiang Liu

**Affiliations:** 1 Department of Cardiology, Xinxiang Central Hospital, Xinxiang, Henan, China; 2 Department of Cardiology, Changzhi People’s Hospital, Changzhi, China; 3 Department of Cardiology, Yunyang County People’s Hospital, Yunyang, China

**Keywords:** Coronary artery disease, heart failure, insulin resistance, metabolic disorders, obesity-metabolic indices

## Abstract

**Background:**

Insulin resistance and metabolic disorders are important mechanisms in heart failure (HF) development. Patients with coronary artery disease (CAD) often present metabolic abnormalities, which may further increase HF risk. Therefore, examining the relationship between obesity-metabolic indices and HF in CAD patients is clinically relevant.

**Methods:**

This multicenter study included 3872 CAD patients. Multivariable logistic regression assessed associations of four obesity-metabolic indices [triglyceride glucose (TyG), TyG-body mass index (TyG-BMI), TyG-waist circumference (TyG-WC), metabolic score for insulin resistance (METS-IR)] with HF. Restricted cubic splines (RCS) evaluated nonlinearity and threshold effects. Diagnostic performance was compared using receiver operating characteristic (ROC), decision curve analysis (DCA), and variable importance measures, with robustness tested via subgroup and sensitivity analyses. Mediation analysis examined renal function as a potential mediator.

**Results:**

All four indices were significantly associated with HF, showing a nonlinear dose-response relationship. Associations strengthened notably when TyG, TyG-BMI, TyG-WC, and METS-IR exceeded 8.27, 240, 520, and 38.5, respectively. Subgroup and sensitivity analyses confirmed robustness. ROC, DCA, and variable importance ranking analyses collectively demonstrated that METS-IR possessed the best diagnostic ability compared to the other three obesity-metabolic indices. Mediation analysis indicated that obesity-metabolic indices may influence HF risk partly through renal function.

**Conclusion:**

Obesity-metabolic indices, particularly METS-IR, are associated with HF in CAD patients and exhibit clear threshold effects. METS-IR demonstrated the strongest diagnostic ability for prevalent HF, and its association with HF was partially mediated by renal function.

## 1 Introduction

Heart failure (HF) represents a clinical syndrome defined by signs and symptoms arising from structural or functional cardiac abnormalities. Diagnosis is typically confirmed through elevated natriuretic peptide levels and/or objective signs of pulmonary or systemic congestion (1–3). HF represents a substantial threat to patients’ health and quality of life (4). The likelihood of developing HF is considerably higher in those with coronary artery disease (CAD). When CAD and HF co-exist, they act together to drive up the rates of disability and mortality. Therefore, a concerted effort to manage CAD patients, specifically to prevent the onset of HF, is particularly crucial (5, 6).

Traditionally, the elevated risk of HF in patients with CAD has often been attributed to factors such as hypertension, poor blood pressure (BP) control, and suboptimal management of blood glucose and lipids (7–9). However, the role of obesity−driven insulin resistance (IR) in this process has not received sufficient attention (10). CAD itself, characterized by atherosclerosis and luminal stenosis, is frequently accompanied by IR and various metabolic disorders, which may collectively exacerbate adverse clinical outcomes including HF (11, 12).

In recent years, the critical role of obesity and IR in the pathogenesis of multiple diseases—such as cardiovascular diseases, sepsis, and cancer—has garnered increasing attention (13–15). Against this backdrop, a series of novel indices reflecting metabolic status have emerged, among which the triglyceride−glucose (TyG) index and its derivatives [TyG-body mass index (TyG-BMI), TyG-waist circumference (TyG-WC)], as well as the metabolic score for IR (METS−IR), are the most commonly cited (16–18). These indices comprehensively reflect the degree of obesity, the state of IR, and the overall metabolic dysregulation, and have demonstrated considerable utility across various diseases (16, 19, 20). For instance, studies have shown that TyG and its derived indices exhibit excellent predictive performance for stroke and cardiovascular mortality (19, 20); in metabolic−associated fatty liver disease, these indices also perform well, suggesting their potential as early screening tools (16). Notably, patients with CAD often present with multiple metabolic abnormalities. These interrelated metabolic disturbances significantly increase the risk of adverse outcomes, among which HF represents one of the most critical complications (21, 22). Therefore, investigating the association between these obesity−metabolic indices and HF in patients with CAD holds important clinical relevance.

This multicenter study was designed to systematically examine the associations between four obesity-metabolic indices—TyG, TyG-BMI, TyG-WC, and METS-IR—and the occurrence of HF in patients with CAD, as well as to explore their potential mediating mechanisms. The findings may provide a reference for weight management, assessment of IR-related metabolic disorders, and early identification of HF risk in this population.

## 2 Materials and methods

### 2.1 Study population

**Figure 1:**
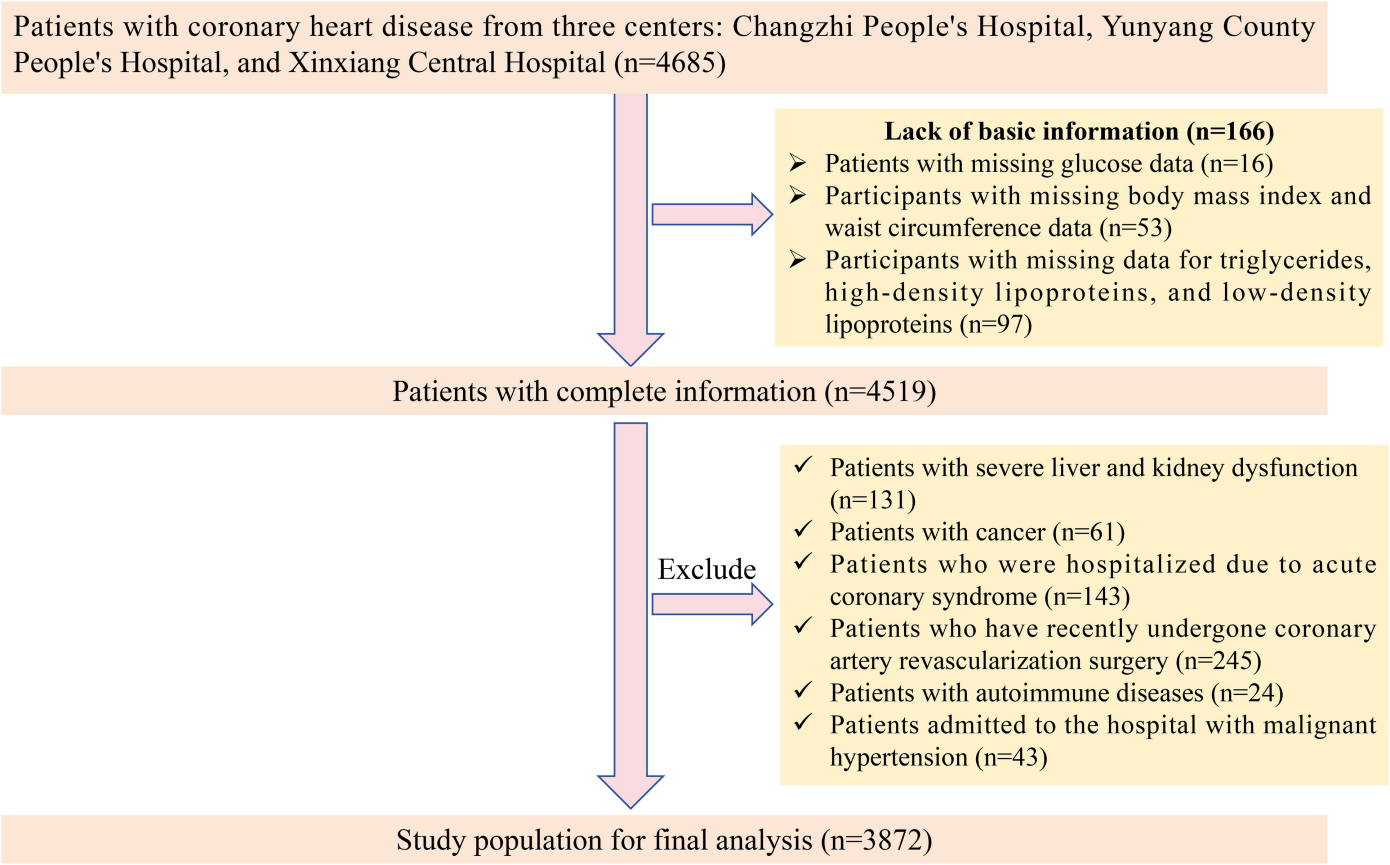
Screening process of the study population.

This was a multicenter retrospective study. Initially, 4685 patients with confirmed CAD were enrolled from three hospitals: Changzhi People’s Hospital in Shanxi Province, Yunyang County People’s Hospital, and Xinxiang Central Hospital. After excluding 166 patients with incomplete baseline information, we further applied the following exclusion criteria: 1) severe hepatic or renal dysfunction; 2) active malignancy; 3) admission due to acute coronary syndrome; 4) recent coronary revascularization; and 5) admission for hypertensive crisis. Following this rigorous screening process, a total of 3872 patients were eligible and included in the final analysis. The detailed patient selection flow is presented in **Figure 1**.

This clinical observational study was conducted in strict accordance with the principles of the Declaration of Helsinki. The study protocol was reviewed and approved by the Ethics Committees of Changzhi People’s Hospital (CZX20180213), Yunyang County People’s Hospital (Yy20200419), and Xinxiang Central Hospital (NXCH20230911). All patients provided informed consent and signed the informed consent form before enrollment.

### 2.2 Collection of covariates and definitions

Data collection for this observational study encompassed the following aspects: 1) Demographic and clinical characteristics, including sex, age, body mass index (BMI), systolic and diastolic BP, smoking status, and alcohol consumption; 2) Laboratory parameters: including liver function, total cholesterol (TC), triglycerides, high-density lipoprotein cholesterol (HDL-C), low-density lipoprotein cholesterol (LDL-C), fasting plasma glucose (FPG), estimated glomerular filtration rate (eGFR), and B-type natriuretic peptide (BNP); 3) Medical history: diabetes, hypertension, and hyperlipidemia; specific diagnostic criteria are provided in the **Supplementary Material**; 4) Medication use, lipid-lowering drugs, antiplatelet drugs, diuretics, beta-blockers, and angiotensin-converting enzyme inhibitors/angiotensin receptor blockers (ACEIs/ARBs).

### 2.3 Calculation of obesity-metabolic indices

Obesity metabolic index, including TyG, TyG-BMI, TyG-WC, and METS-IR. These indices were calculated as follows (16, 17, 23):


TyG = Ln[(triglycerides×fasting glucose)/2]

TyG−BMI = TyG × BMI.

TyG−WC=TyG×WC

METS−IR=[Ln(2×FPG)+Fasting Triglycerides)×BMI]/Ln(HDL−C)

### 2.4 Clinical outcomes

The clinical outcome of interest in this study was the occurrence of HF events. Diagnosis was made in strict accordance with current HF, based on a comprehensive assessment including clinical symptoms, physical signs, and echocardiographic findings (24–26). Detailed diagnostic criteria are provided in the **Supplementary Material**.

### 2.5 Statistical analysis

Participants were divided into two groups according to the presence or absence of HF for inter-group comparisons. Continuous variables are presented as mean±standard deviation (SD) or median (interquartile range) depending on their distribution, while categorical variables are expressed as frequency and percentage. Multivariable-adjusted logistic regression was used to examine the associations between the four obesity-metabolic indices and HF. Restricted cubic splines (RCS) were further applied to assess potential dose-response relationships and identify threshold effects; based on the identified thresholds, piecewise regression models were constructed for two-phase comparative analysis.

To assess the diagnostic utility of each obesity-metabolic index for HF, we utilized receiver operating characteristic (ROC) curves, decision curve analysis (DCA), and variable importance ranking. Subgroup and sensitivity analyses were further performed to test the robustness of the primary outcomes. Mediation analysis was also conducted to examine whether the obesity-metabolic index influences HF onset through specific intermediary pathways. Detailed statistical procedures are described in the **Supplementary Material**.

All analyses were carried out with R software (version 4.4.3), adopting a two-sided P < 0.05 as the threshold for statistical significance.

## 3 Results

### 3.1 Comparison of baseline characteristics between HF and Non-HF groups

Among the 3872 patients with CAD enrolled in this study, 806 were identified as having HF. Based on HF diagnosis, participants were categorized into two groups, with their baseline demographic and clinical features summarized in **Table 1**.

**Table 1:** Basic characteristics of the research population.

Characteristic	Overall	Non-HF group	HF group	p value
Number	3872	3066	806	
Age (years)	65.33±10.93	65.15±11.04	65.99±10.49	0.054
Sex (%)				0.643
Female	2004 (51.76%)	1581 (51.57%)	423 (52.48%)	
Male	1868 (48.24%)	1485 (48.43%)	383 (47.52%)	
BMI (kg/m^2^)	26.17±3.74	26.08±3.55	26.53±4.34	0.002
SBP (mmHg)	144.89±18.04	145.01±18.12	144.43±17.74	0.417
DBP (mmHg)	86.84±13.85	86.59±13.84	87.76±13.88	0.033
Smoking (%)	1072 (27.69%)	870 (28.38%)	202 (25.06%)	0.061
Drinking (%)	889 (22.96%)	688 (22.44%)	201 (24.94%)	0.133
Laboratory tests				
ALT (U/L)	66.00 (57.00-74.00)	66.00 (57.00-74.00)	67.00 (58.00-75.00)	0.287
AST (U/L)	18.00 (15.00-22.00)	18.00 (15.00-22.00)	18.00 (15.00-22.00)	0.101
TC (mmol/L)	4.20±0.90	4.21±0.90	4.18±0.91	0.368
Triglycerides (mmol/L)	0.94 (0.79-1.12)	0.92 (0.78-1.11)	0.97 (0.81-1.13)	0.002
HDL-C (mg/dL)	2.51±0.89	2.52±0.88	2.47±0.92	0.136
LDL-C (mg/dL)	1.16±0.27	1.15±0.27	1.18±0.28	0.022
FPG (mmol/L)	5.28±1.30	5.27±1.27	5.35±1.39	0.135
eGFR (mL/min/1.73 m²)	91.77±22.17	92.42±22.30	89.30±21.54	<0.001
BNP (pg/mL)	30.32 (21.50-38.57)	25.80 (21.16-33.20)	175.82 (170.40-236.96)	<0.001
Obesity-metabolic indices				
TyG	8.28±0.41	8.23±0.40	8.46±0.40	<0.001
TyG-BMI	242.28±40.55	236.38±37.18	264.72±44.79	<0.001
TyG-WC	528.97±104.53	509.23±90.97	604.06±117.92	<0.001
METS-IR	39.14±7.74	37.68±6.73	44.70±8.73	<0.001
Medical history				
Hypertension (%)	1817 (46.93%)	1339 (43.67%)	478 (59.31%)	<0.001
Diabetes (%)	1152 (29.75%)	892 (29.09%)	260 (32.26%)	0.080
Hyperlipidemia (%)	844 (21.80%)	641 (20.91%)	203 (25.19%)	0.032
Medications				
Lipid-lowering drugs	3836 (99.07%)	3033 (98.92%)	803 (99.63%)	0.064
Antiplatelet drugs	3835 (99.04%)	3034 (98.96%)	801 (99.38%)	0.272
Diuretics (%)	1803 (46.57%)	999 (32.58%)	804 (99.75%)	<0.001
Beta-blockers (%)	3800 (98.14%)	2998 (97.78%)	802 (99.50%)	0.001
ACEIs/ARBs (%)	2761 (71.31%)	2100 (68.49%)	661 (82.01%)	<0.001

Data are presented as mean ± standard deviation, median (interquartile range), or as numbers, and percentages.

HF, Heart failure; BMI, body mass index; SBP, systolic blood pressure; DBP, diastolic blood pressure; ALT, alanine transaminase; AST, aspartate transaminase; HDL-C, high-density lipoprotein cholesterol; LDL-C, low-density lipoprotein cholesterol; TC, total cholesterol; FPG, fasting plasma glucose; eGFR, estimated Glomerular Filtration Rate; BNP, Brain Natriuretic Peptide; TyG, Triglyceride-Glucose index; TyG-BMI, TyG combining with body mass index; TyG-WC, TyG combining with waist circumference; METS-IR, Metabolic score for insulin resistance; ACEIs, angiotensin-converting enzyme inhibitors; ARBs, angiotensin receptor blockers.

In terms of general characteristics, the heart failure group had significantly higher BMI and DBP compared to the non-HF group. Laboratory test results showed that TG, LDL-C, eGFR, and BNP levels were also significantly higher in the HF group. Moreover, all four obesity-metabolic indexes were noticeably higher in the HF group. Regarding comorbidities and medication usage, the HF group had a higher prevalence of hypertension and hyperlipidemia. They were also more likely to be taking diuretics, beta-blockers, and ACEIs/ARBs (**Table 1**). No significant differences were observed between the two groups in other aspects.

### 3.2 Relationship between four obesity-metabolic indices and HF in patients with CAD

**Figure 2:**
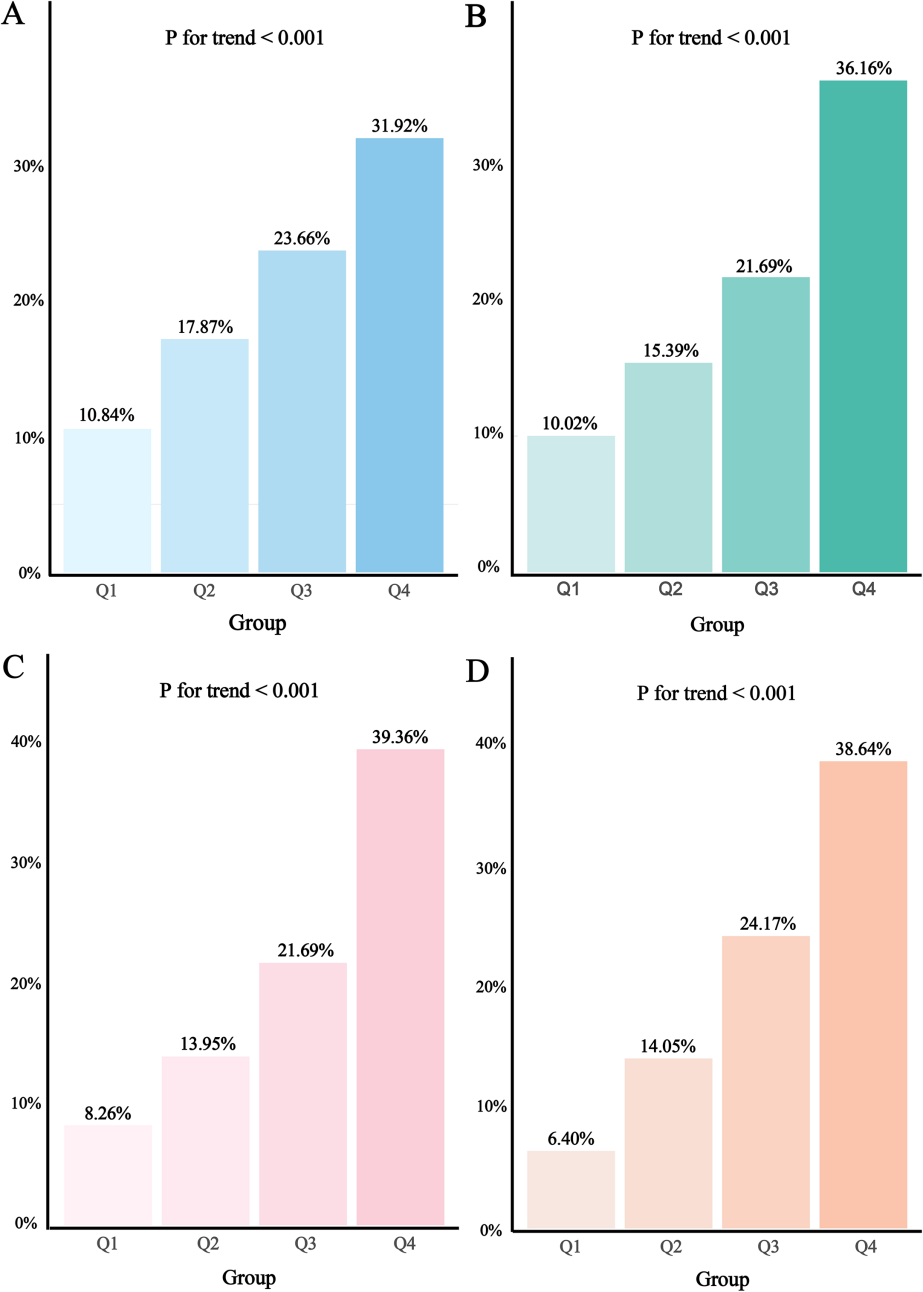
Prevalence of HF among different groups with various obesity-related metabolic indices. **(A)**, TyG; **(B)**, TyG-BMI; **(C)**, TyG-WC; **(D)**, METS-IR.

To investigate the relationship between different obesity-metabolic indices and HF, we first divided the participants into four groups based on the quartiles of the four obesity-metabolic indices and compared the HF prevalence across these groups. The results were clear: for all four indices, the prevalence of HF gradually increased from the Q1 group to the Q4 group (**Figure 2**).

Next, we used logistic regression and established four models to adjust for covariates and assess the relationship between these indices and HF. The results indicated that in the unadjusted Model 1, for each 1 SD increase in the four obesity-metabolic indices—TyG, TyG-BMI, TyG-WC, and METS-IR—the odds of HF increased by 75.6% [odds ratio (OR):1.756, 95% confidence interval (CI):1.620-1.906], 104.8% (OR:2.048, 95%CI: 1.884-2.230), 154.3% (OR:2.543, 95%CI: 2.330-2.782), and 157.7% (OR:2.577, 95%CI: 2.355-2.826), respectively (**Table 2**). In the fully adjusted models, the ORs remained significant, with values of 1.769 (95%CI:1.628-1.924), 2.036 (95% CI: 1.869-2.221), 2.557 (95% CI: 2.355-2.826), and 2.541 (95% CI: 2.327-2.780) for TyG, TyG-BMI, TyG-WC, and METS-IR, respectively (**Table 2**). Additionally, when we converted these indices into categorical variables, the results remained consistent: compared to the Q1 group, the HF risk gradually increased from Q2 to Q4, showing a clear increasing trend (P for trend < 0.001). Specifically, the odds of HF for the Q4 group compared to the Q1 group were 3.981, 5.032, 7.500, and 9.201 times greater for the four indices, respectively (**Table 2**).

**Table 2:** Relationship between different obesity-metabolic indices and HF in patients with CAD.

HF	Model 1	Model 2	Model 3	Model 4
	OR (95% CI) P	OR (95% CI) P	OR (95% CI) P	OR (95% CI) P
TyG				
TyG (per 1SD increase)	1.756 [1.620, 1.906] <0.001	1.779 [1.639, 1.933] <0.001	1.781 [1.641, 1.936] <0.001	1.769 [1.628, 1.924] <0.001
Quartiles of TyG				
Q 1	Reference	Reference	Reference	Reference
Q 2	1.806 [1.383, 2.369] <0.001	1.786 [1.371, 2.337] <0.001	1.785 [1.370, 2.336] <0.001	1.757 [1.351, 2.295] <0.001
Q 3	2.734 [2.119, 3.549] <0.001	2.659 [2.066, 3.442] <0.001	2.659 [2.065, 3.443] <0.001	2.631 [2.048, 3.400] <0.001
Q 4	4.058 [3.170, 5.230] <0.001	4.067 [3.185, 5.230] <0.001	4.079 [3.194, 5.248] <0.001	3.981 [3.124, 5.109] <0.001
P for trend	<0.001	<0.001	<0.001	<0.001
TyG-BMI				
TyG**-**BMI (per 1SD increase)	2.048 [1.884, 2.230] <0.001	2.037 [1.872, 2.220] <0.001	2.040 [1.875, 2.224] <0.001	2.036 [1.869, 2.221] <0.001
Quartiles of TyG-BMI				
Q 1	Reference	Reference	Reference	Reference
Q 2	1.670 [1.268, 2.208] <0.001	1.634 [1.246, 2.151] <0.001	1.623 [1.236, 2.141] <0.001	1.628 [1.239, 2.148] <0.001
Q 3	2.537 [1.955, 3.314] <0.001	2.488 [1.924, 3.236] <0.001	2.498 [1.929, 3.254] <0.001	2.485 [1.918, 3.238] <0.001
Q 4	5.064 [3.950, 6.542] <0.001	5.085 [3.986, 6.540] <0.001	5.045 [3.947, 6.501] <0.001	5.032 [3.936, 6.486] <0.001
P for trend	<0.001	<0.001	<0.001	<0.001
TyG-WC				
TyG-WC (per 1SD increase)	2.543 [2.330, 2.782] <0.001	2.575 [2.356, 2.821] <0.001	2.578 [2.358, 2.824] <0.001	2.577 [2.355, 2.826] <0.001
Quartiles of TyG-WC				
Q 1	Reference	Reference	Reference	Reference
Q 2	1.800 [1.342, 2.429] <0.001	1.799 [1.346, 2.418] <0.001	1.796 [1.342, 2.418] <0.001	1.797 [1.342, 2.420] <0.001
Q 3	3.095 [2.349, 4.113] <0.001	3.075 [2.345, 4.068] <0.001	3.081 [2.345, 4.084] <0.001	3.091 [2.352, 4.099] <0.001
Q 4	7.363 [5.661, 9.677] <0.001	7.205 [5.569, 9.422] <0.001	7.502 [5.784, 9.837] <0.001	7.500 [5.780, 9.835] <0.001
P for trend	<0.001	<0.001	<0.001	<0.001
METS-IR				
METS-IR (per 1SD increase)	2.577 [2.355, 2.826] <0.001	2.575 [2.356, 2.821] <0.001	2.543 [2.330, 2.782] <0.001	2.541 [2.327, 2.780] <0.001
Quartiles of METS-IR				
Q 1	Reference	Reference	Reference	Reference
Q 2	2.442 [1.784, 3.376] <0.001	2.368 [1.734, 3.267] <0.001	2.384 [1.745, 3.290] <0.001	2.389 [1.752, 3.291] <0.001
Q 3	4.755 [3.545, 6.465] <0.001	4.731 [3.534, 6.420] <0.001	4.746 [3.545, 6.442] <0.001	4.659 [3.486, 6.312] <0.001
Q 4	9.205 [6.922, 12.425] <0.001	9.201 [6.936, 12.391] <0.001	9.227 [6.952, 12.432] <0.001	9.201 [6.948, 12.370] <0.001
P for trend	<0.001	<0.001	<0.001	<0.001

Model 1: no covariates were adjusted. .

Model 2: age, sex, BMI, smoking status and drinking status were adjusted.

Model 3: Model 2 plus adjustment for SBP, DBP, ALT, AST, TC, triglyceride, HDL.C, LDL.C, BNP, FPG, DM, Hyperlipidemia and Hypertension.

Model 4: Model 3 plus adjustment for The usage of Lipid-lowering drugs, Antiplatelet medication, Diuretics, Beta-blockers, Calcium channel blockers, and ACEIs/ARBs.

CAD, coronary artery disease; HF, Heart failure; TyG, Triglyceride-Glucose index; TyG-BMI, TyG combining with body mass index; TyG-WC, TyG combining with waist circumference; METS-IR, Metabolic score for insulin resistance, OR, Odds Ratio; CI, confidence interval.

Other abbreviations, see **Table 1**.

### 3.3 Dose-response and threshold analysis of obesity-metabolic indices and HF in patients with CAD

**Figure 3:**
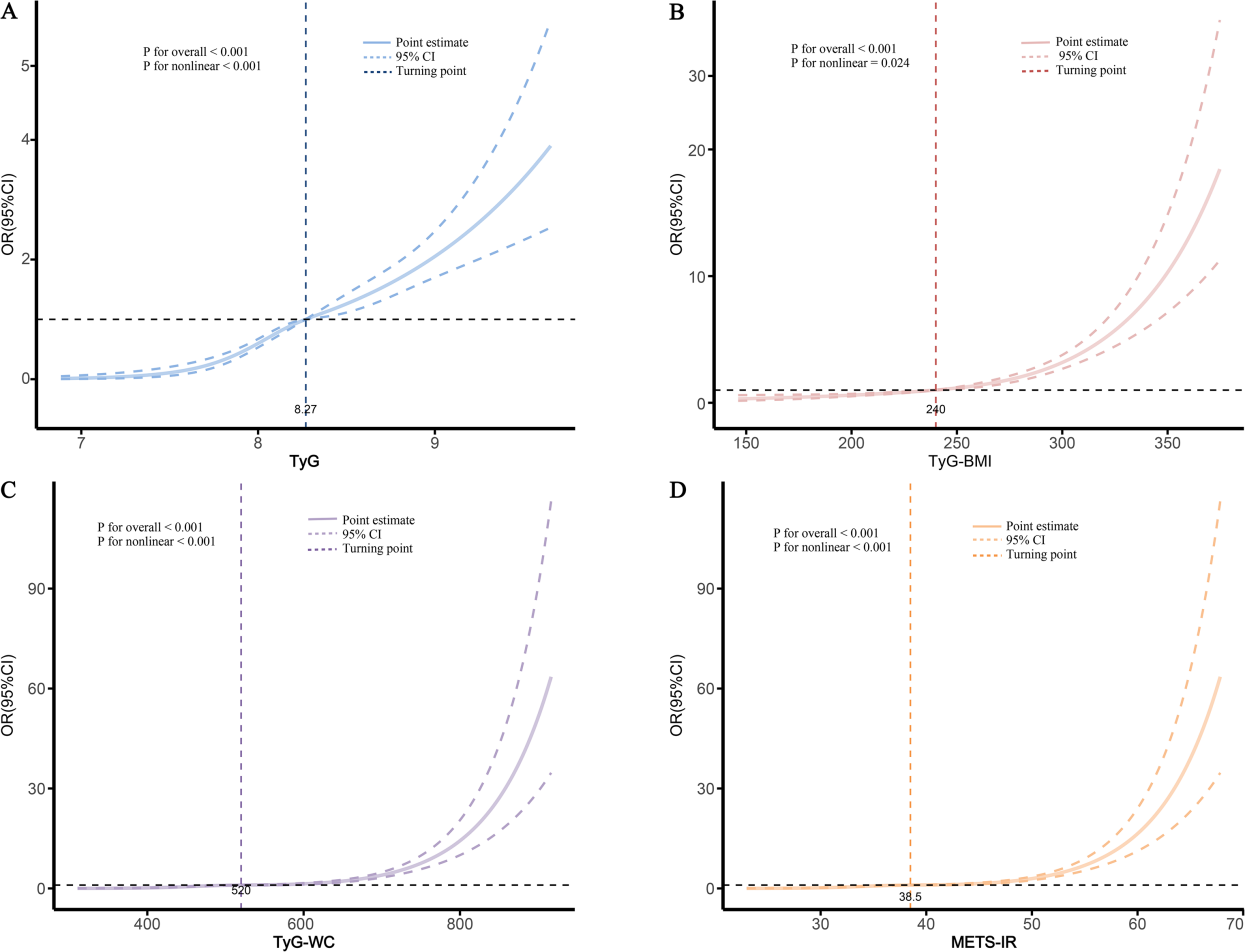
Dose-response relationship between various obesity-related metabolic indices and HF. **(A)**, TyG; **(B)**, TyG-BMI; **(C)**, TyG-WC; **(D)**, METS-IR.

To further explore the dose-response relationship between the four obesity-metabolic indices and HF, we performed analysis using RCS. The results indicated that all four indices exhibited a nonlinear relationship with HF (P < 0.05) (**Figure 3**). Specifically, the risk of HF significantly increased when the values of the four indices—TyG, TyG-BMI, TyG-WC, and METS-IR—exceeded 8.27, 240, 520, and 38.5, respectively (**Figure 3**).

Based on the thresholds identified by the RCS analysis, we performed a threshold analysis. The results showed that compared to patients with values below or equal to the threshold, patients with TyG, TyG-BMI, TyG-WC, and METS-IR exceeding the identified thresholds of 8.27, 240, 520, and 38.5 had risks 2.444 (95%CI: 2.076-2.884), 2.759 (95%CI: 2.334-3.269), 4.027 (95%CI: 3.378-4.817), and 4.028 (95%CI: 3.379-4.819) times higher, respectively (**Table 3**, **Figure 1**). This further underscores the strong association between elevated obesity-metabolic indices and the increased risk of HF in patients with CAD. Moreover, exceeding these thresholds significantly amplifies the risk. These findings highlight that controlling weight and reducing insulin resistance-related metabolic disturbances may help decrease the incidence of HF events.

**Table 3:** Two-stage comparative analysis based on the turning point of RCS.

HF	Model 1 OR (95% CI) P	Model 2 OR (95% CI) P	Model 3 OR (95% CI) P	Model 4 OR (95% CI) P
TyG				
Turning point	8.27	8.27	8.27	8.27
<= 8.27	Reference	Reference	Reference	Reference
> 8.27	2.459 [2.085, 2.906] <0.001	2.433 [2.069, 2.867] <0.001	2.426 [2.061, 2.860] <0.001	2.444 [2.076, 2.884] <0.001
TyG-BMI				
Turning point (ng/dL)	240	240	240	240
<= 240	Reference	Reference	Reference	Reference
> 240	2.788 [2.365, 3.293] <0.001	2.810 [2.382, 3.324] <0.001	2.770 [2.347, 3.278] <0.001	2.759 [2.334, 3.269] <0.001
TyG-WC				
Turning point (ng/dL)	520	520	520	520
<= 520	Reference	Reference	Reference	Reference
> 520	4.072 [3.420, 4.865] <0.001	4.047 [3.401, 4.831] <0.001	4.030 [3.391, 4.807] <0.001	4.027 [3.378, 4.817] <0.001
METS-IR				
Turning point (ng/dL)	38.5	38.5	38.5	38.5
<= 38.5	Reference	Reference	Reference	Reference
> 38.5	4.076 [3.422, 4.867] <0.001	4.048 [3.403, 4.832] <0.001	4.032 [3.392, 4.809] <0.001	4.028 [3.379, 4.819] <0.001

Model 1: no covariates were adjusted.

Model 2: age, sex, BMI, smoking status and drinking status were adjusted.

Model 3: Model 2 plus adjustment for SBP, DBP, ALT, AST, TC, triglyceride, HDL.C, LDL.C, BNP, FPG, DM, Hyperlipidemia and Hypertension.

Model 4: Model 3 plus adjustment for The usage of Lipid-lowering drugs, Antiplatelet medication, Diuretics, Beta-blockers, Calcium channel blockers, and ACEIs/ARBs.

HF, Heart failure; RCS, Restrictive Cubic Spline; TyG, Triglyceride-Glucose index; TyG-BMI, TyG combining with body mass index; TyG-WC, TyG combining with waist circumference; METS-IR, Metabolic score for insulin resistance, OR, Odds Ratio; CI, confidence interval.

Other abbreviations, see **Table 1**.

### 3.4 Subgroup analysis and sensitivity analysis

**Figure 4:**
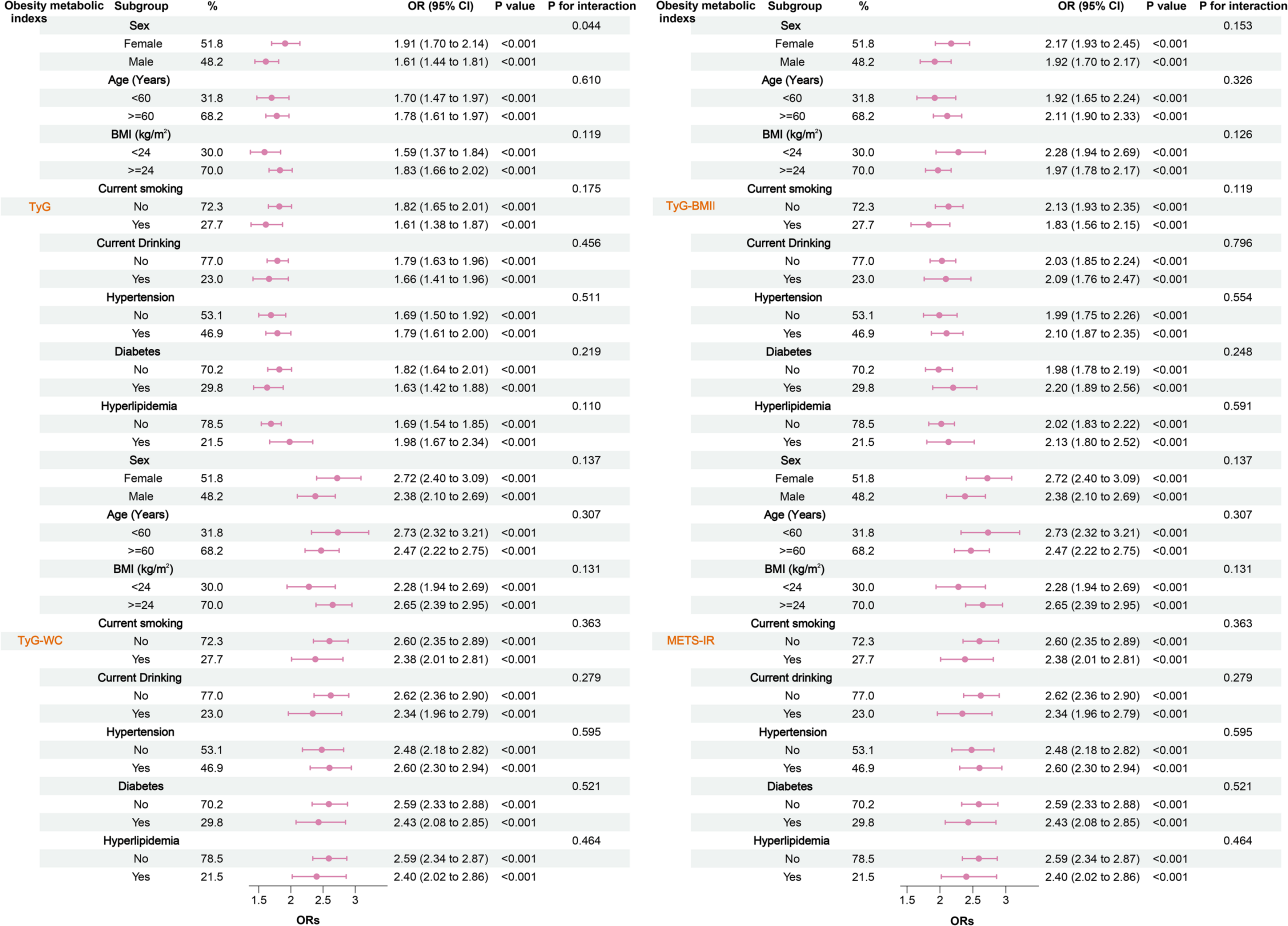
Relationship between various obesity-related metabolic indices and HF in different subgroups.

Considering that factors such as patients’ baseline characteristics, lifestyle habits, and disease status might influence the study results, we conducted subgroup analyses based on gender, age, BMI, smoking status, alcohol consumption, hypertension, diabetes, and hyperlipidemia. As shown in **Figure 4**, all four obesity-metabolic indices remained significantly associated with HF risk across all subgroups, indicating that these relationships are independent of the stratification variables.

To further evaluate the robustness of the main results, we performed multiple sensitivity analyses. Initially, we performed a subgroup analysis excluding patients with acute HF, as this population typically presents with more severe complications and complex metabolic disturbances that could confound the association between obesity-related metabolic indices and HF. After exclusion, the results remained materially consistent with those from the overall study population, indicating that the inclusion of acute HF patients did not significantly influence our primary findings (**Table 1**). Subsequently, to reduce potential confounding from advanced age, we excluded participants older than 75 years. The associations did not change materially (**Table 2**). Furthermore, given the pathophysiological differences between HF subtypes, we further examined the relationship between obesity-related metabolic indices and specific HF subtypes. The results demonstrated that elevated levels of these indices were significantly associated with both HF subtypes (**Table 3**). Finally, to account for potential residual confounding factors, we conducted an E-value analysis for unmeasured confounders. The results indicated that unmeasured confounding factors would need to be exceptionally large to overturn our findings (**Table 4**).

**Table 4:** ROC curves of four obesity-metabolic indices were compared and analyzed to evaluate their ability to diagnose HF events in patients with CAD.

HF	AUC	95%CI low	95%CI up	Specificity	Sensitivity	Positive-pv	Negative-pv
TyG	0.656	0.635	0.676	0.536	0.710	0.287	0.875
TyG-BMI	0.684	0.663	0.705	0.538	0.733	0.294	0.885
TyG-WC	0.731	0.712	0.750	0.667	0.675	0.348	0.886
METS-IR	0.751	0.732	0.769	0.676	0.690	0.373	0.886

TyG, triglyceride glucose; TyG-BMI, TyG-body mass index;  TyG-WC, TyG-waist circumference; METS-IR, metabolic score for insulin resistance; CAD, coronary artery disease; AUC, area under the curve; Positive-pv, positive predictive value; Negative-pv, negative predictive value.

Other abbreviations, see **Table 1**.

### 3.5 Comparison of the diagnostic performance of different obesity-metabolic indices for HF

**Figure 5:**
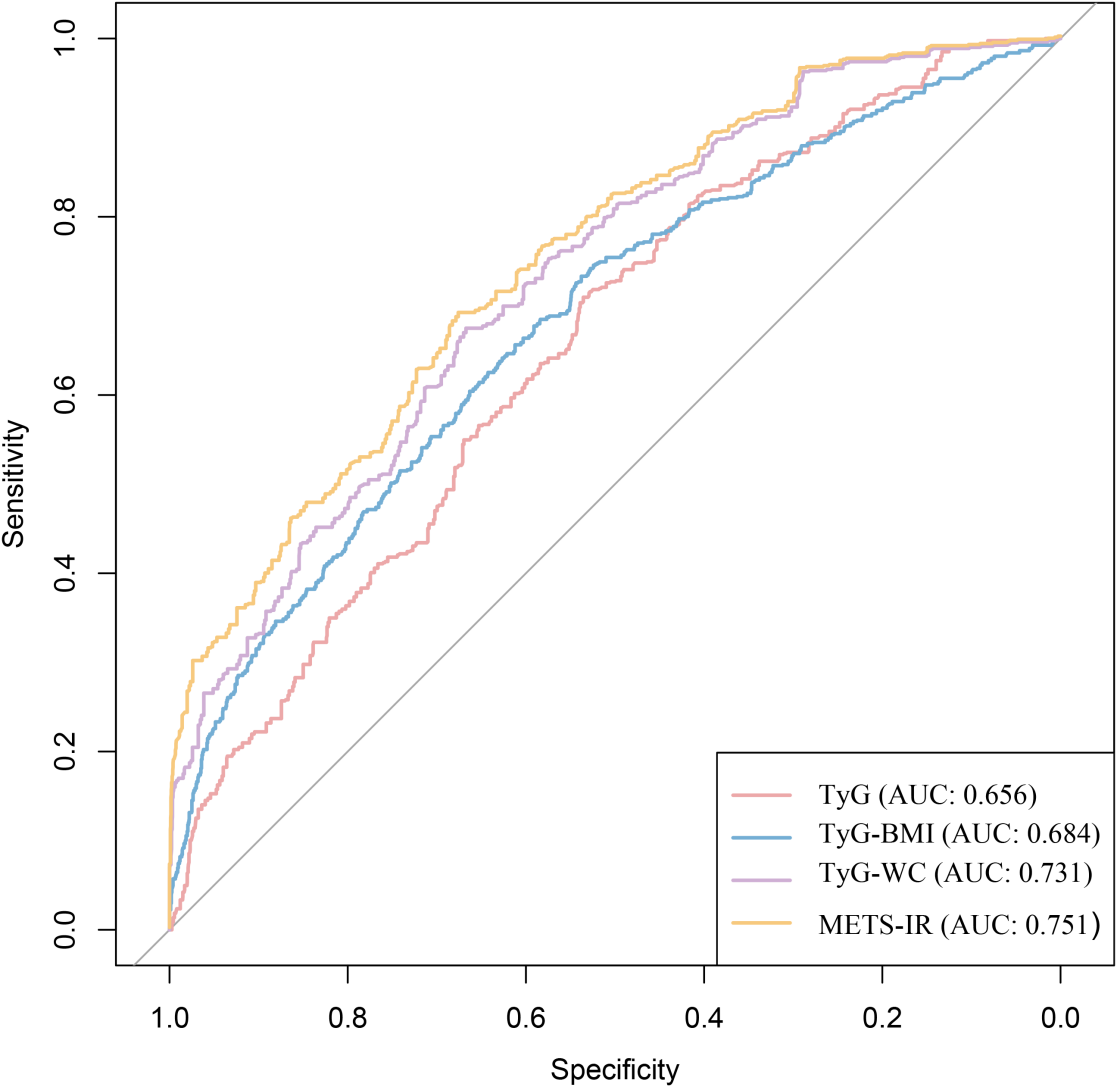
Receiver operating characteristic curve analysis of various obesity-related metabolic indices for the diagnostic performance of HF.

Considering that all four obesity-metabolic indices were closely associated with the occurrence of HF, we performed a series of comparative analyses to identify the best diagnostic indicator. First, we compared the indices using ROC curves. The results showed that all four indices demonstrated good diagnostic performance, with the area under the curve (AUC) for TyG, TyG-BMI, TyG-WC, and METS-IR all exceeding 0.65. Specifically, the AUCs were 0.656, 0.684, 0.731, and 0.751, respectively (**Table 4**, **Figure 5**). Among them, METS-IR showed the best diagnostic ability, with both sensitivity and specificity being relatively good (**Table 4**, **Figure 5**).

**Figure 6:**
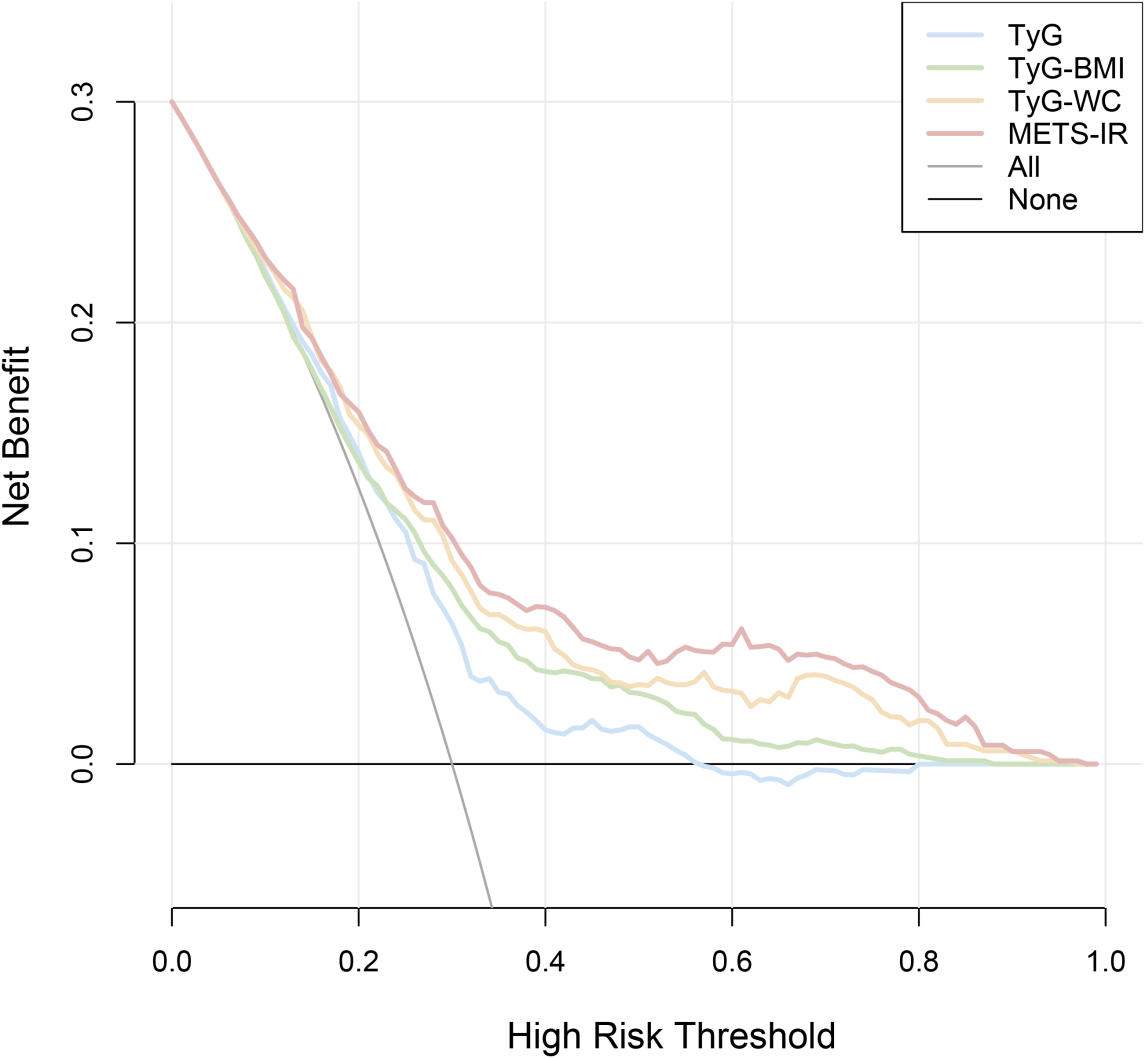
Decision curve analysis of various obesity-related metabolic indices for the diagnostic performance of HF.

**Figure 7:**
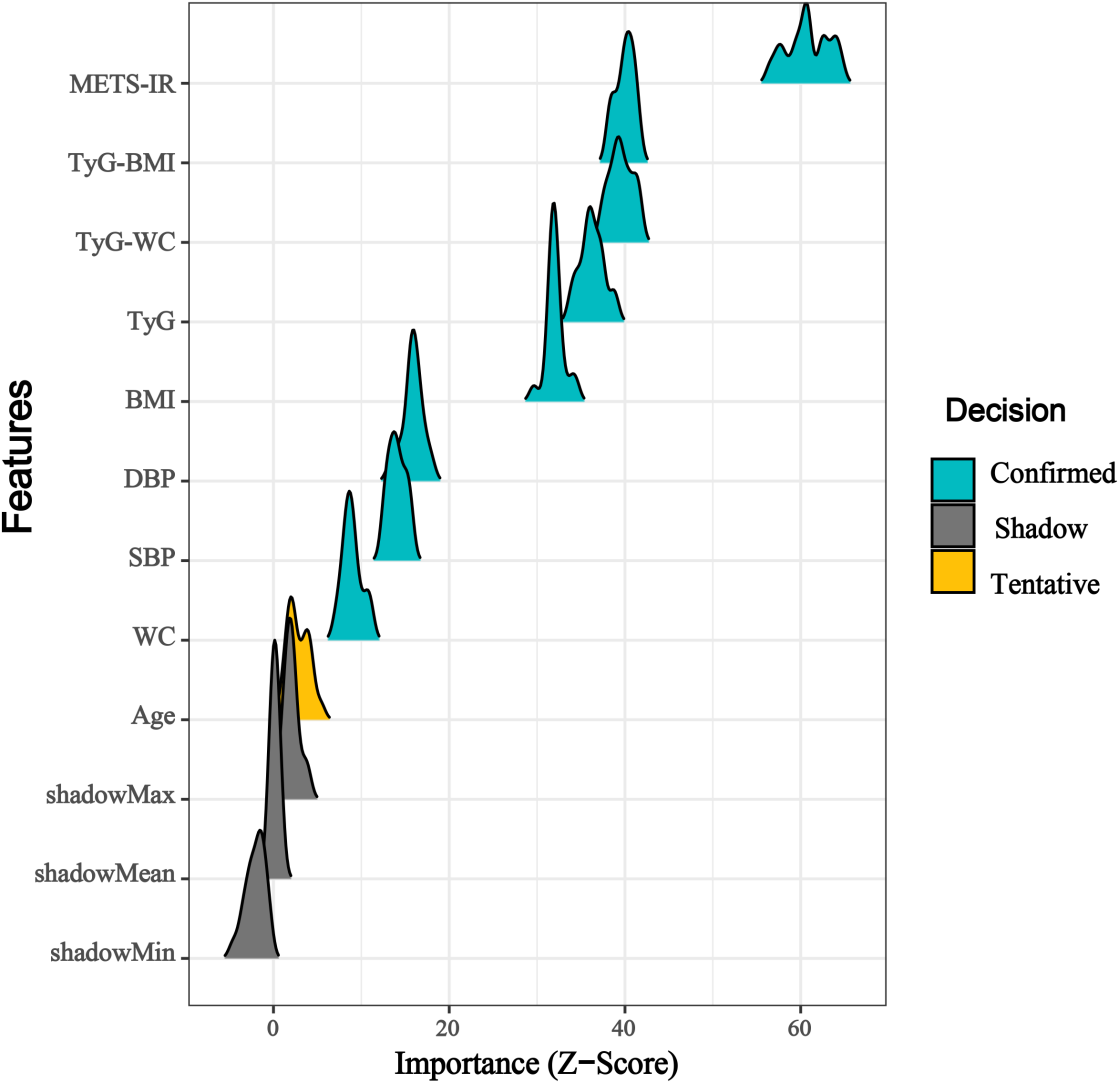
Feature importance ridge plot based on Boruta.

To further assess the clinical utility of these indices, we performed DCA to evaluate their net benefit. The results consistently indicated that METS-IR provided the highest net benefit compared to the other three obesity-metabolic indices (**Figure 6**). Additionally, we employed the Boruta algorithm to determine the variable importance of different indices in machine learning models. The findings were consistent with our previous analyses, demonstrating that METS-IR had the highest variable importance among all variables (**Figure 7**). Finally, we compared the diagnostic capability of the obesity-metabolic indices with that of the traditional HF scoring system. The results revealed that METS-IR continued to outperform the traditional HF scoring system in diagnosing HF (**Figure 2**).

### 3.6 Role of renal function in mediation

**Figure 8:**
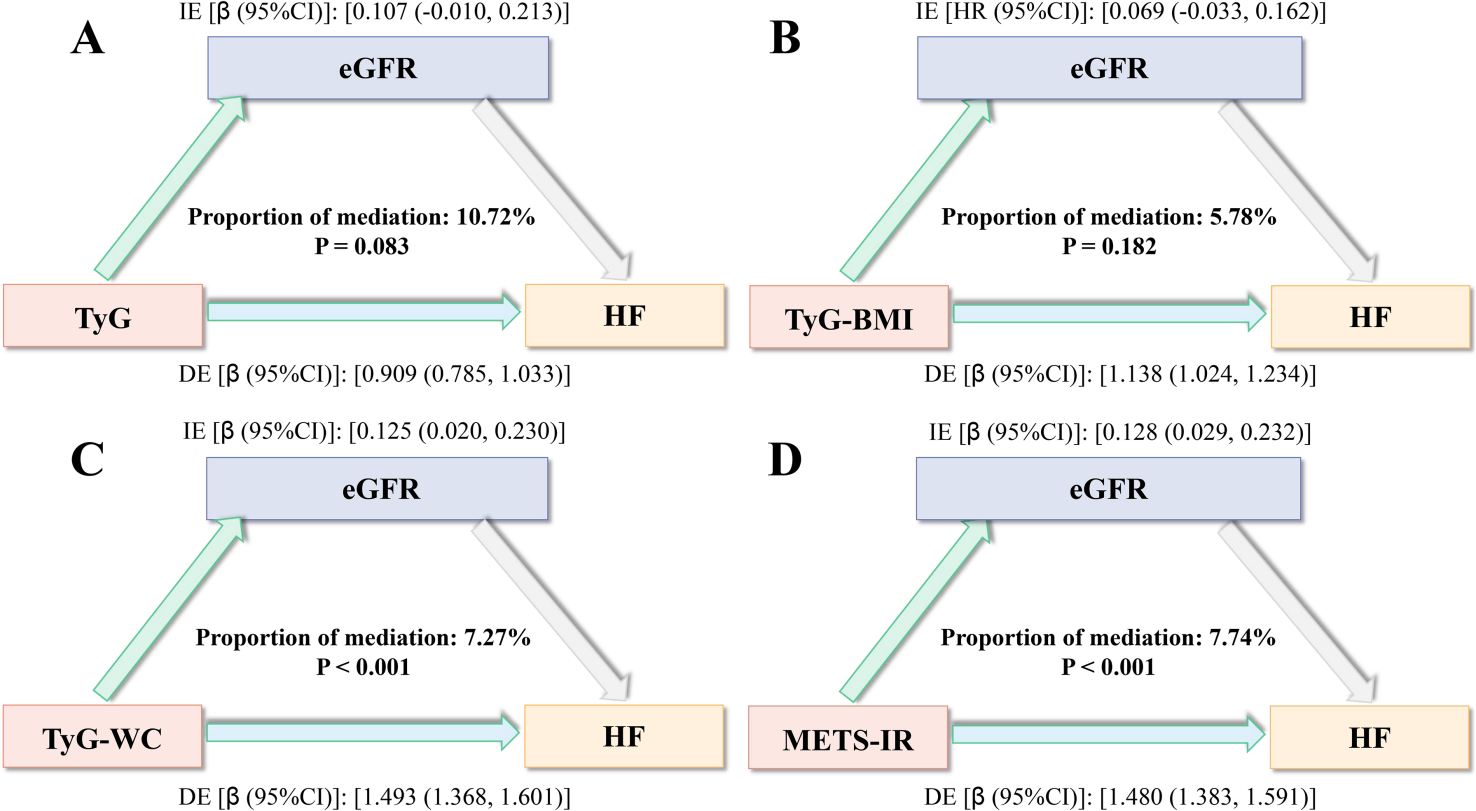
Role of renal function in mediation. **(A)**, TyG; **(B)**, TyG-BMI; **(C)**, TyG-WC; **(D)**, METS-IR.IE, Indirect effect; DE, Direct effect.

To further explore the potential pathways through which obesity-metabolic indices may influence the occurrence of HF, we screened relevant variables and identified that eGFR likely plays a key mediating role (**Figure 8**). Mediation analysis revealed that the four obesity-metabolic indices—particularly TyG-WC and METS-IR—partially mediated the development of HF by affecting eGFR, which reflects renal function, with mediation proportions of 7.27% and 7.74%, respectively (**Figure 8**). These findings suggest that obesity-metabolic indices may contribute to HF at least in part through the pathway of renal function impairment.

Additionally, given the close relationship between obesity, metabolic disorders, and chronic inflammation, we further investigated whether systemic inflammatory indicators mediate the association between obesity-metabolic indices and HF. The results demonstrated that METS-IR also partially mediated the occurrence of HF by influencing inflammatory markers, indicating that inflammation plays a significant role in this pathway (**Figure 3**). This finding underscores the potential importance of anti-inflammatory strategies in mitigating obesity-related HF risk.

Collectively, these mediation analyses suggest that both renal function impairment and systemic inflammation may serve as important intermediary mechanisms linking obesity-metabolic indices to the development of HF.

## 4 Discussion

Current evidence has established that obesity-metabolic indices such as TyG, TyG-BMI, TyG-WC, and METS-IR are associated with various adverse outcomes including cardiovascular mortality, autoimmune diseases, sepsis, and cancer (23, 27–29). However, their relationship with HF in patients with CAD has not been sufficiently studied. Our multicenter study systematically demonstrates a significant association between these four indices and HF in patients with CAD, with a clear threshold effect observed. In particular, when TyG, TyG-BMI, TyG-WC, and METS-IR exceed 8.27, 240, 520, and 38.5 respectively, their association with HF becomes significantly stronger. Further analysis indicated that all four obesity-metabolic indices possess diagnostic value for HF, with METS-IR demonstrating the most outstanding overall performance. Mediation analysis further suggested that these indices, particularly METS-IR, may contribute to HF occurrence partly through impairing renal function. In conclusion, our findings highlight METS-IR as a superior obesity-metabolic indicator that not only assists in weight management and metabolic assessment in high-risk CAD populations but may also offer important clinical insights for early HF prevention through improving IR and preserving renal function.

Patients with CAD represent a large, high-risk population, with a global prevalence exceeding 200 million. CAD remains a leading cause of mortality worldwide (4). These individuals endure a multifaceted burden, including persistent symptoms such as angina and dyspnea, reduced quality of life, and a significantly elevated risk for major adverse cardiovascular events, such as myocardial infarction and stroke (30–32). A critical long-term complication of CAD is its progression to HF, with studies showing that nearly two-thirds of HF cases have underlying CAD (33, 34). Key modifiable risk factors, including hypertension, dyslipidemia, diabetes, and smoking, contribute to both the progression of CAD and the onset of HF (35, 36). Consequently, comprehensive management is crucial. This includes aggressive risk factor control, guideline-directed medical therapy such as SGLT2 inhibitors and ARNIs, and vigilant monitoring for early signs of HF decompensation, all of which are vital for improving survival and reducing the significant associated mortality (6, 37–39).

Obesity-metabolic indices are increasingly being applied in the diagnosis and prediction of a growing number of diseases (40–42). A recent meta-analysis on the TyG index and CAD found that patients with a high TyG index have a 7.95 times higher risk of developing CAD compared to those with a low TyG index. This suggests that individuals with elevated TyG levels face significantly increased risks of developing CAD and stroke (43). In a Japanese cross−sectional study, each 10−unit rise in TyG−BMI was associated with a 31% increase in hypertension prevalence, confirming a significant relationship between TyG−BMI and hypertension (44). Analyses based on the National Health and Nutrition Examination Survey (NHANES) database further demonstrated that a 1−unit increment in TyG−WC elevated the risk of cardiac death by 1.45−fold and diabetes−related mortality by 2.545−fold among individuals with metabolic syndrome (45). Another NHANES−derived study reported that METS−IR was correlated with both all−cause and cardiovascular mortality, with a notably higher risk of all−cause mortality observed when METS−IR exceeded 41.33 (46). Furthermore, a growing body of recent evidence underscores that the type of obesity, rather than just its presence, may exert differential effects on cardiovascular disease risk (47, 48). It has been consistently demonstrated that central fat distribution, as opposed to peripheral adiposity, serves as a stronger predictor of cardiometabolic complications (47). This suggests that the adverse metabolic and clinical impact is more closely linked to visceral fat accumulation than to overall or peripheral adiposity. These collective findings imply that IR and metabolic disturbances reflected by obesity−related indices may contribute to the pathogenesis of multiple conditions, including HF. This highlights the clinical relevance of weight control, obesity mitigation, and the management of IR−related metabolic dysregulation.

METS-IR integrates multiple indicators reflecting obesity and metabolism, including FPG, triglycerides, BMI, and HDL-C. These metabolic parameters are closely associated with various diseases, including HF. Compared to single indicators, METS-IR, by combining these parameters, may more accurately reflect the overall metabolic status and IR level in the body. Therefore, its comprehensive nature likely contributes to its superior diagnostic capability in identifying HF. In the future, this simple and easily accessible tool may potentially be used for HF risk assessment and high-risk population screening among patients with CAD.

The progression from CAD to HF is critically accelerated by underlying metabolic disturbances, primarily obesity and IR (49–51). These conditions create a synergistic pathological environment through several interconnected mechanisms. First, IR disrupts myocardial substrate metabolism, shifting energy production toward less efficient fatty acid oxidation and promoting the intracellular accumulation of toxic lipid intermediates. This accumulation induces cardiomyocyte apoptosis and mitochondrial dysfunction (49, 51, 52). Second, dysfunctional visceral adipose tissue acts as an endocrine organ, secreting pro-inflammatory adipokines such as TNF-α and IL-6, which foster a state of chronic low-grade systemic inflammation (53–55). This inflammation contributes to coronary microvascular endothelial dysfunction and promotes direct myocardial fibrosis (56, 57). Third, hyperinsulinemia and activation of the sympathetic nervous system lead to sodium retention, increased plasma volume, and hypertension, creating a chronic pressure overload that results in left ventricular hypertrophy and diastolic dysfunction (58–60). Collectively, these processes—metabolic derangement, inflammation, and neurohormonal activation—compromise cardiac structure and function, thereby facilitating the development of HF, particularly heart failure with preserved ejection fraction, in patients with established CAD (61, 62).

The main innovations of this study are as follows: First, the multicenter design enhances the generalizability of the findings to a broader population. Second, this study is the first to systematically demonstrate, in a high-risk CAD cohort, that all four obesity-metabolic indices—TyG, TyG-BMI, TyG-WC, and METS-IR—are significantly associated with HF and exhibit clear threshold effects, providing a valuable reference range for future risk stratification. Finally, comparative analyses revealed that METS-IR possesses the best diagnostic performance for HF in CAD patients, and mediation analysis further suggested that its association with HF may be partly mediated through renal function. This provides a rationale for weight management and improvement of IR to preserve renal function and potentially reduce HF risk.

This study also has several limitations. First, since our study only included patients from the Chinese population, caution should be exercised when generalizing our findings to other populations, regions, or ethnic groups. Further validation studies in diverse geographic regions and different ethnic populations are warranted. Second, all obesity-metabolic indices were calculated based on data collected during the first hospitalization, and their dynamic changes and potential influence on HF could not be assessed; future studies should explore this aspect further. Thirdly, given the inherent nature of observational studies, causal inference is not feasible. Therefore, the findings of the current study should be interpreted as demonstrating an association between the obesity-metabolic indices and HF, and causal relationships can only be established through large-scale prospective cohort studies or rigorous randomized controlled trials in the future. Fourth, although extensive confounding factors were adjusted for, unmeasured confounding may still exist; however, E-value analysis suggested that such influence on the main conclusions is likely limited.

## 5 Conclusion

This study found that all four obesity-metabolic indices were significantly associated with HF in patients with CAD, exhibiting a clear threshold effect. Notably, when METS-IR exceeded 38.5, its association with HF was significantly enhanced. Moreover, METS-IR demonstrated superior diagnostic performance and may promote HF through renal function impairment. Of course, as this was an observational study, inherent limitations such as potential reverse causality preclude definitive conclusions regarding causal relationships. Therefore, large-scale, multicenter, prospective cohort studies or randomized controlled trials focusing on metabolic and weight-loss interventions are warranted to further validate and extend our findings.

## Data Availability

The raw data supporting the conclusions of this article will be made available by the authors, without undue reservation.
