# Supplementary material for: Association of obesity-metabolic indices with heart failure in patients with coronary artery disease: a multicenter retrospective observational study
Source: Front Endocrinol (Lausanne). 2026 Apr 17;17:1826249. doi: 10.3389/fendo.2026.1826249 (PMC13132743; doi:10.3389/fendo.2026.1826249)
Supplement: Supplementary file 1 [file DataSheet1.docx]

Supplementary Material

# Supplemental material and methods

Baseline examination

Anthropometric measurements were taken by trained nurses. Data for height, weight, and waist circumference (WC) were acquired following a protocol standardized to an accuracy of 0.1 kg and 0.1 cm, respectively. During the measurements, participants were asked to wear light clothing, no hats, and no shoes. At the end of normal expiration, WC was measured with an inelastic tape from the midpoint between the lower ribs and the upper edge of the iliac crest to the nearest 0.1 cm. For laboratory measurements: All biochemical indicators, including triglycerides, glucose, and other lipid profiles, were measured using automated biochemical analyzers in the clinical laboratory of each respective center, following standardized operating procedures. Current smokers were defined as having smoked 100 cigarettes in their lifetime and currently smoking. Alcohol consumption was evaluated with questions regarding the types of alcoholic beverages, the frequency of alcohol consumption per week, and the usual amount consumed per occasion. Subjects who reported alcohol consumption >140 g/week for men and >70 g/week for women were deemed to have excessive alcohol consumption. The body mass index (BMI) was calculated as per the formula: Weight (kg)/Height^2^ (m).

Definitions

Criteria for hypertension included self-reported hypertension, current use of anti-hypertensive medication, or systolic blood pressure (SBP) ≥ 140 mmHg and/or diastolic blood pressure (DBP) ≥ 90 mmHg recorded for at least three consecutive readings. diabetes was defined as fasting serum glucose ≥7.0 mmol/L, the 2-h serum glucose of the oral glucose tolerance test ≥11.1 mmol/L, or the current use of hypoglycaemic medication or insulin. Coronary artery disease (CAD) was defined as the presence of fatal or non-fatal myocardial infarction, unstable angina, and/or coronary revascularization procedures. These diagnoses were further confirmed and established using coronary angiography. Hyperlipidemia is defined as an abnormal elevation of lipid levels in the bloodstream, primarily characterized by increased concentrations of total cholesterol (TC), low-density lipoprotein cholesterol (LDL-C), and triglycerides (TG), or a reduction in high-density lipoprotein cholesterol (HDL-C). According to international guidelines, hyperlipidemia is diagnosed when TC levels exceed 6.2 mmol/L (240 mg/dL), LDL-C levels are ≥ 4.1 mmol/L (160 mg/dL), TG levels are ≥ 2.3 mmol/L (200 mg/dL), or HDL-C levels fall below 1.0 mmol/L (40 mg/dL) in men and 1.3 mmol/L (50 mg/dL) in women.

Diagnostic Criteria for Heart Failure (HF) with Reduced Ejection Fraction (HFrEF)

HFrEF was diagnosed based on the following criteria:

1. Presence of typical signs and/or symptoms of heart failure;
2. Symptoms: dyspnea, fatigue, reduced exercise tolerance, orthopnea;
3. Signs: peripheral edema, pulmonary rales, elevated jugular venous pressure;
4. Objective evidence of cardiac dysfunction: Left ventricular ejection fraction (LVEF) ≤ 40% measured by transthoracic echocardiography using the biplane Simpson’s method;
5. Supporting evidence of structural heart abnormality (at least one of the following):

I. Left ventricular hypertrophy (LVH) or left atrial enlargement;

II. Diastolic dysfunction assessed by echocardiography.

**Sample size calculation:** Since this is a cross-sectional observational study, we conducted a post hoc sample size estimation based on the 28% prevalence of heart failure observed among the study subjects. Using the parameters of a two-sided α value of 0.05, a margin of error of 0.02, and a statistical power of 95%, we calculated that the required sample size was 1937 patients. Our final study included 3872 patients with coronary artery disease, which fully met the required sample size requirements and ensured that our analysis had sufficient statistical power.

**Regarding missing data:** For the missing data in other covariates, we used the MICE package in R for multiple imputation. The distribution of the imputed data was consistent with the trend of the original data.

Details of the statistical analyses.

Variables of baseline characteristics are shown as n (%) if categorical, mean (SD) if normally distributed, and median (interquartile range) if nonnormally distributed. To compare the characteristics among obesity metabolic indices groups, the chi-square test was performed for categorical variables, and one-way analysis of variance, or the Kruskal-Wallis test, was performed for continuous variables with normal and skewed distributions.

The association between obesity-metabolic indices and HF in patients with CHD was tested with multivariable Logiatic regression models. This study set four different models (Model 1: unadjusted; Model 2: age, sex, BMI, smoking status and drinking status were adjusted; Model 3: Model 2 plus adjustment for SBP, DBP, ALT, AST, TC, TG, HDL.C, LDL.C, BNP, FPG, DM, Hyperlipidemia and Hypertension. Model 4: Model 3 plus adjustment for The usage of Lipid-lowering drugs, Antiplatelet medication, Diuretics, Beta-blockers, Calcium channel blockers, and ACEIs/ARBs.) to adjust. Tests for trend were conducted, assigning the median value within each tertile to the corresponding tertile. In addition, the restricted inverse square spline (four nodes at the 5th, 35th, 65th, and 95th percentiles of the obesity-metabolic indices distribution) was used to evaluate the nonlinear relationships. Finally, receiver operating characteristic curves, Decision curve analysis, and Boruta feature importance were applied to compare the diagnostic performance of these markers. Finally, an intermediary analysis was conducted to explore whether other factors mediated the occurrence of HF. The mediation analysis should satisfy the condition that the mediator variable, the exposure variable, and the outcome variable all have significant statistical significance.

All analyses were done using R (version 4.4.3). All P-values were two-sided, and P-values of <0.05 denoted statistical significance.

1. Supplementary Tables

**Table S1**. Relationship between different obesity-metabolic indices and HF in patients with CAD after excluding patients with acute HF

| HF | Model 1 | Model 2 | Model 3 | Model 4 |
| --- | --- | --- | --- | --- |
|  | OR (95% CI) P | OR (95% CI) P | OR (95% CI) P | OR (95% CI) P |
| TyG |  |  |  |  |
| TyG (per 1SD increase) | 1.757 [1.616, 1.912] <0.001 | 1.762 [1.619, 1.919] <0.001 | 1.769 [1.625, 1.928] <0.001 | 1.754 [1.611, 1.913] <0.001 |
| Quartiles of TyG |  |  |  |  |
| Q 1 | Reference | Reference | Reference | Reference |
| Q 2 | 1.824 [1.384, 2.415] <0.001 | 1.846 [1.399, 2.447] <0.001 | 1.843 [1.396, 2.446] <0.001 | 1.869 [1.413, 2.485] <0.001 |
| Q 3 | 2.708 [2.082, 3.547] <0.001 | 2.682 [2.059, 3.518] <0.001 | 2.694 [2.067, 3.536 <0.001 | 2.784 [2.131, 3.662 <0.001 |
| Q 4 | 4.030 [3.124, 5.242] <0.001 | 4.078 [3.155, 5.314] <0.001 | 4.107 [3.175, 5.357] <0.001 | 4.088 [3.154, 5.341] <0.001 |
| P for trend | <0.001 | <0.001 | <0.001 | <0.001 |
| TyG-BMI |  |  |  |  |
| TyG**-**BMI (per 1SD increase) | 2.026 [1.858, 2.213] <0.001 | 2.037 [1.866, 2.226 <0.001 | 2.034 [1.863, 2.224] <0.001 | 2.034 [1.861, 2.226] <0.001 |
| Quartiles of TyG**-**BMI |  |  |  |  |
| Q 1 | Reference | Reference | Reference | Reference |
| Q 2 | 1.566 [1.182, 2.083] 0.002 | 1.566 [1.181, 2.086] 0.002 | 1.588 [1.196, 2.117] 0.001 | 1.625 [1.221, 2.170] <0.001 |
| Q 3 | 2.361 [1.810, 3.100] <0.001 | 2.362 [1.808, 3.106] <0.001 | 2.331 [1.782, 3.067] <0.001 | 2.377 [1.814, 3.134] <0.001 |
| Q 4 | 4.863 [3.783, 6.305] <0.001 | 4.962 [3.853, 6.445] <0.001 | 4.937 [3.830, 6.417] <0.001 | 4.971 [3.848, 6.476] <0.001 |
| P for trend | <0.001 | <0.001 | <0.001 | <0.001 |
| TyG-WC |  |  |  |  |
| TyG-WC (per 1SD increase) | 2.487 [2.273, 2.726] <0.001 | 2.497 [2.279, 2.741] <0.001 | 2.501 [2.282, 2.747 <0.001 | 2.494 [2.274, 2.742] <0.001 |
| Quartiles of  TyG-WC |  |  |  |  |
| Q 1 | Reference | Reference | Reference | Reference |
| Q 2 | 1.732 [1.294, 2.332] <0.001 | 1.716 [1.280, 2.313] <0.001 | 1.733 [1.292, 2.337 <0.001 | 1.728 [1.285, 2.335] <0.001 |
| Q 3 | 2.665 [2.021, 3.542] <0.001 | 2.644 [2.002, 3.519] <0.001 | 2.670 [2.021, 3.556] <0.001 | 2.676 [2.021, 3.572] <0.001 |
| Q 4 | 6.316 [4.868, 8.280 <0.001 | 6.342 [4.878, 8.332] <0.001 | 6.342 [4.875, 8.338] <0.001 | 6.243 [4.787, 8.226] <0.001 |
| P for trend | <0.001 | <0.001 | <0.001 | <0.001 |
| METS-IR |  |  |  |  |
| METS-IR (per 1SD increase) | 2.494 [2.274, 2.742] <0.001 | 2.501 [2.282, 2.747] <0.001 | 2.497 [2.279, 2.741] <0.001 | 2.487 [2.273, 2.726] <0.001 |
| Quartiles of METS-IR |  |  |  |  |
| Q 1 | Reference | Reference | Reference | Reference |
| Q 2 | 2.344 [1.705, 3.256] <0.001 | 2.374 [1.725, 3.302 <0.001 | 2.404 [1.746, 3.346] <0.001 | 2.485 [1.801, 3.465] <0.001 |
| Q 3 | 4.414 [3.277, 6.032 <0.001 | 4.411 [3.269, 6.036 <0.001 | 4.444 [3.291, 6.085] <0.001 | 4.501 [3.327, 6.174<0.001 |
| Q 4 | 8.505 [6.374, 11.529] <0.001 | 8.667 [6.484, 11.768] <0.001 | 8.745 [6.535, 11.885] <0.001 | 8.744 [6.521, 11.908] <0.001 |
| P for trend | <0.001 | <0.001 | <0.001 | <0.001 |

Model 1: no covariates were adjusted.

Model 2: age, sex, BMI, smoking status and drinking status were adjusted.

Model 3: Model 2 plus adjustment for SBP, DBP, ALT, AST, TC, TG, HDL.C, LDL.C, BNP, FPG, DM, Hyperlipidemia and Hypertension.

Model 4: Model 3 plus adjustment for The usage of Lipid-lowering drugs, Antiplatelet medication, Diuretics, Beta-blockers, Calcium channel blockers, and ACEIs/ARBs.

Abbreviations: CAD, coronary artery disease; HF, Heart failure; TyG, Triglyceride-Glucose index; TyG-BMI, TyG combining with body mass index; TyG-WC, TyG combining with waist circumference; METS-IR, Metabolic score for insulin resistance,

OR, Odds Ratio; CI, confidence interval

Other abbreviations, see Table 1.

**Table S2**. Relationship between different obesity-metabolic indices and HF in patients with CAD after excluding patients with age > 75 years

| HF | Model 1 | Model 2 | Model 3 | Model 4 |
| --- | --- | --- | --- | --- |
|  | OR (95% CI) P | OR (95% CI) P | OR (95% CI) P | OR (95% CI) P |
| TyG |  |  |  |  |
| TyG (per 1SD increase) | 1.755 [1.613, 1.910] <0.001 | 1.760 [1.618, 1.918] <0.001 | 1.767 [1.623, 1.927] <0.001 | 1.751 [1.599, 1.912] <0.001 |
| Quartiles of TyG |  |  |  |  |
| Q 1 | Reference | Reference | Reference | Reference |
| Q 2 | 1.822 [1.382, 2.414] <0.001 | 1.843 [1.396, 2.445] <0.001 | 1.841 [1.394, 2.445] <0.001 | 1.864 [1.410, 2.481] <0.001 |
| Q 3 | 2.704 [2.076, 3.543] <0.001 | 2.680 [2.056, 3.516] <0.001 | 2.690 [2.064, 3.533] <0.001 | 2.781 [2.128, 3.658] <0.001 |
| Q 4 | 4.026 [3.122, 5.240] <0.001 | 4.075 [3.151, 5.312] <0.001 | 4.105 [3.174, 5.355] <0.001 | 4.085 [3.152, 5.338] <0.001 |
| P for trend | <0.001 | <0.001 | <0.001 | <0.001 |
| TyG-BMI |  |  |  |  |
| TyG**-**BMI (per 1SD increase) | 2.024 [1.856, 2.212] <0.001 | 2.034 [1.863, 2.224] <0.001 | 2.031 [1.860, 2.222] <0.001 | 2.031 [1.858, 2.224] <0.001 |
| Quartiles of TyG**-**BMI |  |  |  |  |
| Q 1 | Reference | Reference | Reference | Reference |
| Q 2 | 1.564 [1.180, 2.081] 0.002 | 1.564 [1.180, 2.084] 0.002 | 1.586 [1.195, 2.116] 0.001 | 1.621 [1.218, 2.166] <0.001 |
| Q 3 | 2.358 [1.807, 3.098] <0.001 | 2.358 [1.805, 3.104] <0.001 | 2.327 [1.780, 3.065] <0.001 | 2.374 [1.812, 3.131] <0.001 |
| Q 4 | 4.861 [3.782, 6.303] <0.001 | 4.957 [3.848, 6.441] <0.001 | 4.934 [3.827, 6.415] <0.001 | 4.968 [3.845, 6.474] <0.001 |
| P for trend | <0.001 | <0.001 | <0.001 | <0.001 |
| TyG-WC |  |  |  |  |
| TyG-WC (per 1SD increase) | 2.485 [2.271, 2.724] <0.001 | 2.491 [2.273, 2.737] <0.001 | 2.491 [2.275, 2.741] <0.001 | 2.491 [2.272, 2.740] <0.001 |
| Quartiles of  TyG-WC |  |  |  |  |
| Q 1 | Reference | Reference | Reference | Reference |
| Q 2 | 1.728 [1.290, 2.328] <0.001 | 1.711 [1.273, 2.307] <0.001 | 1.728 [1.283, 2.332] <0.001 | 1.722 [1.281, 2.331] <0.001 |
| Q 3 | 2.661 [2.018, 3.537] <0.001 | 2.634 [1.994, 3.516] <0.001 | 2.668 [2.015, 3.552] <0.001 | 2.673 [2.018, 3.569] <0.001 |
| Q 4 | 6.311 [4.862, 8.273] <0.001 | 6.338 [4.872, 8.328] <0.001 | 6.337 [4.872, 8.334] <0.001 | 6.237 [4.782, 8.221] <0.001 |
| P for trend | <0.001 | <0.001 | <0.001 | <0.001 |
| METS-IR |  |  |  |  |
| METS-IR (per 1SD increase) | 2.491 [2.272, 2.741] <0.001 | 2.493 [2.278, 2.744] <0.001 | 2.495 [2.276, 2.739] <0.001 | 2.482 [2.268, 2.722] <0.001 |
| Quartiles of METS-IR |  |  |  |  |
| Q 1 | Reference | Reference | Reference | Reference |
| Q 2 | 2.341 [1.703, 3.254] <0.001 | 2.371 [1.723, 3.329] <0.001 | 2.403 [1.745, 3.344] <0.001 | 2.481 [1.796, 3.462] <0.001 |
| Q 3 | 4.409 [3.272, 6.029] <0.001 | 4.408 [3.266, 6.033] <0.001 | 4.441 [3.289, 6.083] <0.001 | 4.496 [3.325, 6.171]<0.001 |
| Q 4 | 8.501 [6.372, 11.526] <0.001 | 8.664 [6.482, 11.765] <0.001 | 8.740 [6.532, 11.883] <0.001 | 8.738 [6.517, 11.904] <0.001 |
| P for trend | <0.001 | <0.001 | <0.001 | <0.001 |

Model 1: no covariates were adjusted.

Model 2: age, sex, BMI, smoking status and drinking status were adjusted.

Model 3: Model 2 plus adjustment for SBP, DBP, ALT, AST, TC, TG, HDL.C, LDL.C, BNP, FPG, DM, Hyperlipidemia and Hypertension.

Model 4: Model 3 plus adjustment for The usage of Lipid-lowering drugs, Antiplatelet medication, Diuretics, Beta-blockers, Calcium channel blockers, and ACEIs/ARBs.

Abbreviations: CAD, coronary artery disease; HF, Heart failure; TyG, Triglyceride-Glucose index; TyG-BMI, TyG combining with body mass index; TyG-WC, TyG combining with waist circumference; METS-IR, Metabolic score for insulin resistance,

OR, Odds Ratio; CI, confidence interval

Other abbreviations, see Table 1.

**Table S3**. Relationship between different obesity-related metabolic indicators and the subtypes of HF in patients with CAD

| Subtypes of HF | Model 1 | Model 2 | Model 3 | Model 4 |
| --- | --- | --- | --- | --- |
|  | OR (95% CI) P | OR (95% CI) P | OR (95% CI) P | OR (95% CI) P |
| HFrEF |  |  |  |  |
| TyG |  |  |  |  |
| TyG (per 1SD increase) | 1.633 [1.503, 1.776] <0.001 | 1.638 [1.506, 1.782] <0.001 | 1.641 [1.510, 1.786] <0.001 | 1.626 [1.495, 1.770 <0.001 |
| Quartiles of TyG |  |  |  |  |
| Q 1 | Reference | Reference | Reference | Reference |
| Q 2 | 1.804 [1.366, 2.395] <0.001 | 1.783 [1.351, 2.364] <0.001 | 1.793 [1.357, 2.381] <0.001 | 1.815 [1.372, 2.414] <0.001 |
| Q 3 | 2.450 [1.876, 3.221] <0.001 | 2.460 [1.885, 3.231] <0.001 | 2.458 [1.881, 3.233] <0.001 | 2.516 [1.922, 3.314] <0.001 |
| Q 4 | 3.759 [2.907, 4.900] <0.001 | 3.723 [2.882, 4.849] <0.001 | 3.778 [2.920, 4.927] <0.001 | 3.746 [2.891, 4.893] <0.001 |
| P for trend | <0.001 | <0.001 | <0.001 | <0.001 |
| TyG-BMI |  |  |  |  |
| TyG**-**BMI (per 1SD increase) | 2.024 [1.855, 2.212] <0.001 | 2.022 [1.853, 2.209] <0.001 | 2.020 [1.853, 2.206] <0.001 | 2.011 [1.842, 2.199] <0.001 |
| Quartiles of TyG**-**BMI |  |  |  |  |
| Q 1 | Reference | Reference | Reference | Reference |
| Q 2 | 1.642 [1.228, 2.205] <0.001 | 1.613 [1.208, 2.163] <0.001 | 1.597 [1.198, 2.140] <0.001 | 1.590 [1.192, 2.132] <0.001 |
| Q 3 | 2.273 [1.724, 3.017] <0.001 | 2.245 [1.705, 2.975] <0.001 | 2.245 [1.707, 2.972 <0.001 | 2.238 [1.700, 2.966] <0.001 |
| Q 4 | 4.906 [3.787, 6.417] <0.001 | 4.937 [3.816, 6.447] <0.001 | 4.871 [3.771, 6.351] <0.001 | 4.922 [3.806, 6.425] <0.001 |
| P for trend | <0.001 | <0.001 | <0.001 | <0.001 |
| TyG-WC |  |  |  |  |
| TyG-WC (per 1SD increase) | 2.174 [1.994, 2.375] <0.001 | 2.168 [1.989, 2.367] <0.001 | 2.166 [1.988, 2.363] <0.001 | 2.165 [1.985, 2.366] <0.001 |
| Quartiles of  TyG-WC |  |  |  |  |
| Q 1 | Reference | Reference | Reference | Reference |
| Q 2 | 1.839 [1.357, 2.509] <0.001 | 1.832 [1.350, 2.502] <0.001 | 1.830 [1.347, 2.504] <0.001 | 1.818 [1.340, 2.482 <0.001 |
| Q 3 | 2.749 [2.061, 3.702] <0.001 | 2.742 [2.054, 3.697] <0.001 | 2.727 [2.039, 3.681] <0.001 | 2.729 [2.045, 3.678] <0.001 |
| Q 4 | 6.475 [4.942, 8.588] <0.001 | 6.531 [4.976, 8.676] <0.001 | 6.369 [4.844, 8.474] <0.001 | 6.534 [4.980, 8.677] <0.001 |
| P for trend | <0.001 | <0.001 | <0.001 | <0.001 |
| METS-IR |  |  |  |  |
| METS-IR (per 1SD increase) | 2.166 [1.988, 2.363] <0.001 | 2.174 [1.994, 2.375] <0.001 | 2.168 [1.989, 2.367 <0.001 | 2.165 [1.985, 2.366] <0.001 |
| Quartiles of METS-IR |  |  |  |  |
| Q 1 | Reference | Reference | Reference | Reference |
| Q 2 | 2.394 [1.719, 3.373 <0.001 | 2.453 [1.759, 3.461] <0.001 | 2.350 [1.689, 3.308] <0.001 | 2.363 [1.698, 3.328] <0.001 |
| Q 3 | 4.297 [3.151, 5.952] <0.001 | 4.309 [3.156, 5.977] <0.001 | 4.270 [3.135, 5.908] <0.001 | 4.268 [3.131, 5.910] <0.001 |
| Q 4 | 8.917 [6.616, 12.232 <0.001 | 8.871 [6.572, 12.185] <0.001 | 8.675 [6.447, 11.880] <0.001 | 8.766 [6.509, 12.017] <0.001 |
| P for trend | <0.001 | <0.001 | <0.001 | <0.001 |
| HFpEF |  |  |  |  |
| TyG |  |  |  |  |
| TyG (per 1SD increase) | 1.823 [1.539, 2.153] <0.001 | 1.809 [1.525, 2.140] <0.001 | 1.807 [1.523, 2.138] <0.001 | 1.789 [1.509, 2.118] <0.001 |
| Quartiles of TyG |  |  |  |  |
| Q 1 | Reference | Reference | Reference | Reference |
| Q 2 | 1.392 [0.682, 2.918] 0.367 | 1.389 [0.680, 2.919] 0.372 | 1.384 [0.677, 2.904] 0.377 | 1.373 [0.672, 2.884] 0.388 |
| Q 3 | 2.838 [1.534, 5.586] 0.001 | 2.817 [1.518, 5.561] 0.002 | 2.744 [1.481, 5.411] 0.002 | 2.756 [1.487, 5.434] 0.002 |
| Q 4 | 3.498 [1.927, 6.802] <0.001 | 3.429 [1.883, 6.684] <0.001 | 3.457 [1.901, 6.731] <0.001 | 3.478 [1.912, 6.774] <0.001 |
| P for trend | <0.001 | <0.001 | <0.001 | <0.001 |
| TyG-BMI |  |  |  |  |
| TyG**-**BMI (per 1SD increase) | 1.515 [1.269, 1.804] <0.001 | 1.503 [1.260, 1.790 <0.001 | 1.494 [1.252, 1.780] <0.001 | 1.483 [1.243, 1.766] <0.001 |
| Quartiles of TyG**-**BMI |  |  |  |  |
| Q 1 | Reference | Reference | Reference | Reference |
| Q 2 | 1.732 [0.851, 3.679] 0.137 | 1.703 [0.838, 3.614] 0.149 | 1.681 [0.828, 3.559] 0.158 | 1.671 [0.822, 3.544] 0.164 |
| Q 3 | 3.294 [1.758, 6.633 <0.001 | 3.250 [1.736, 6.540] <0.001 | 3.255 [1.742, 6.540] <0.001 | 3.239 [1.731, 6.517 <0.001 |
| Q 4 | 3.533 [1.897, 7.086] <0.001 | 3.538 [1.903, 7.087] <0.001 | 3.524 [1.899, 7.047] <0.001 | 3.554 [1.912, 7.118] <0.001 |
| P for trend | <0.001 | <0.001 | <0.001 | <0.001 |
| TyG-WC |  |  |  |  |
| TyG-WC (per 1SD increase) | 2.522 [2.148, 2.970] <0.001 | 2.464 [2.097, 2.903] <0.001 | 2.463 [2.095, 2.905] <0.001 | 2.461 [2.095, 2.900] <0.001 |
| Quartiles of  TyG-WC |  |  |  |  |
| Q 1 | Reference | Reference | Reference | Reference |
| Q 2 | 1.349 [0.568, 3.325] 0.501 | 1.345 [0.565, 3.318] 0.505 | 1.338 [0.564, 3.291] 0.512 | 1.331 [0.560, 3.279] 0.519 |
| Q 3 | 4.244 [2.127, 9.428] <0.001 | 4.239 [2.120, 9.430] <0.001 | 4.235 [2.126, 9.395] <0.001 | 4.220 [2.115, 9.374] <0.001 |
| Q 4 | 5.976 [3.074, 13.060] <0.001 | 5.843 [2.997, 12.794] <0.001 | 6.172 [3.183, 13.462] <0.001 | 5.992 [3.083, 13.093] <0.001 |
| P for trend | <0.001 | <0.001 | <0.001 | <0.001 |
| METS-IR |  |  |  |  |
| METS-IR (per 1SD increase) | 2.522 [2.148, 2.970 <0.001 | 2.464 [2.097, 2.903] <0.001 | 2.463 [2.095, 2.905] <0.001 | 2.461 [2.095, 2.900 <0.001 |
| Quartiles of METS-IR |  |  |  |  |
| Q 1 | Reference | Reference | Reference | Reference |
| Q 2 | 2.367 [1.055, 5.804] 0.034 | 2.304 [1.028, 5.646] 0.041 | 2.292 [1.023, 5.614] 0.043 | 2.274 [1.016, 5.564] 0.045 |
| Q 3 | 4.926 [2.408, 11.438] <0.001 | 4.928 [2.411, 11.433] <0.001 | 4.908 [2.402, 11.384] <0.001 | 5.038 [2.470, 11.672] <0.001 |
| Q 4 | 6.016 [2.976, 13.867] <0.001 | 6.114 [3.028, 14.083] <0.001 | 6.001 [2.974, 13.815] <0.001 | 5.987 [2.972, 13.766] <0.001 |
| P for trend | <0.001 | <0.001 | <0.001 | <0.001 |

Model 1: no covariates were adjusted.

Model 2: age, sex, BMI, smoking status and drinking status were adjusted.

Model 3: Model 2 plus adjustment for SBP, DBP, ALT, AST, TC, TG, HDL.C, LDL.C, BNP, FPG, DM, Hyperlipidemia and Hypertension.

Model 4: Model 3 plus adjustment for The usage of Lipid-lowering drugs, Antiplatelet medication, Diuretics, Beta-blockers, Calcium channel blockers, and ACEIs/ARBs.

Abbreviations: CAD, coronary artery disease; HF, Heart failure; TyG, Triglyceride-Glucose index; TyG-BMI, TyG combining with body mass index; TyG-WC, TyG combining with waist circumference; METS-IR, Metabolic score for insulin resistance,

OR, Odds Ratio; CI, confidence interval; HFrEF, Heart Failure with Reduced Ejection Fraction; HFpEF, Heart Failure with Preserved Ejection Fraction

Other abbreviations, see Table 1.

**Table S4** E-values for the observed associations between various obesity-metabolic indices and HF.

| Exposure | Adjusted model | E-value |
| --- | --- | --- |
|  | OR (95% CI) |  |
| TyG (per SD increase) | 1.769 [1.628, 1.924] | 2.935 |
| TyG-BMI (per SD increase) | 2.036 [1.869, 2.221] | 3.488 |
| TyG-WC (per SD increase) | 2.577 [2.355, 2.826] | 4.593 |
| METS-IR (per SD increase) | 2.541 [2.327, 2.780] | 4.520 |

The observed associations are the fully adjusted odds ratios (95% confidence intervals) shown in Tables 2 and 3 and are presented here for reference.

3 Supplementary Figures


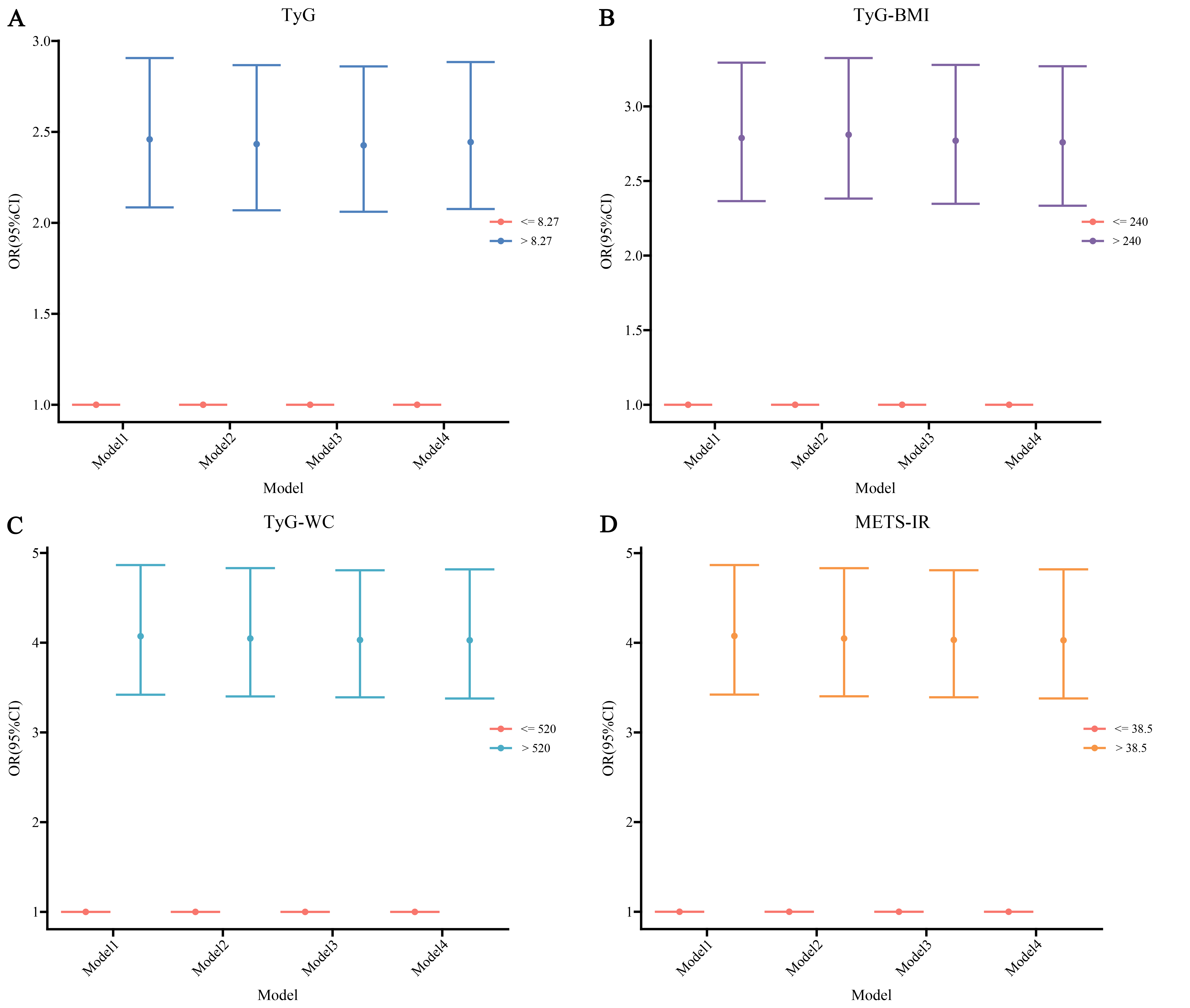


**Figure S1.** Threshold benefits of different obesity-related metabolic indices

(A), TyG; (B), TyG-BMI; (C), TyG-WC; (D), METS-IR


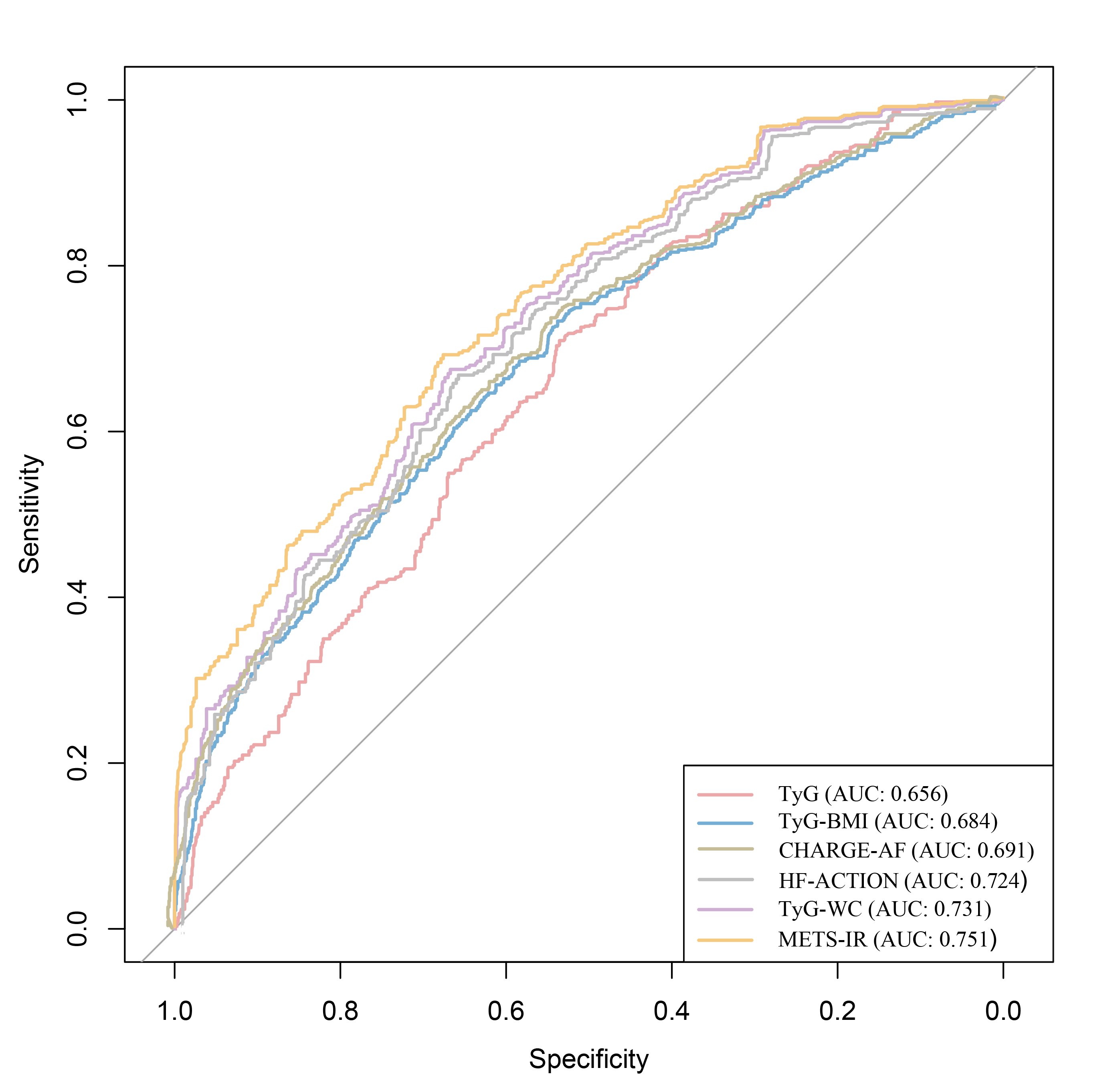


**Figure S2.** Comparison of the diagnostic ability for HF between obesity-metabolic indices and traditional HF scores


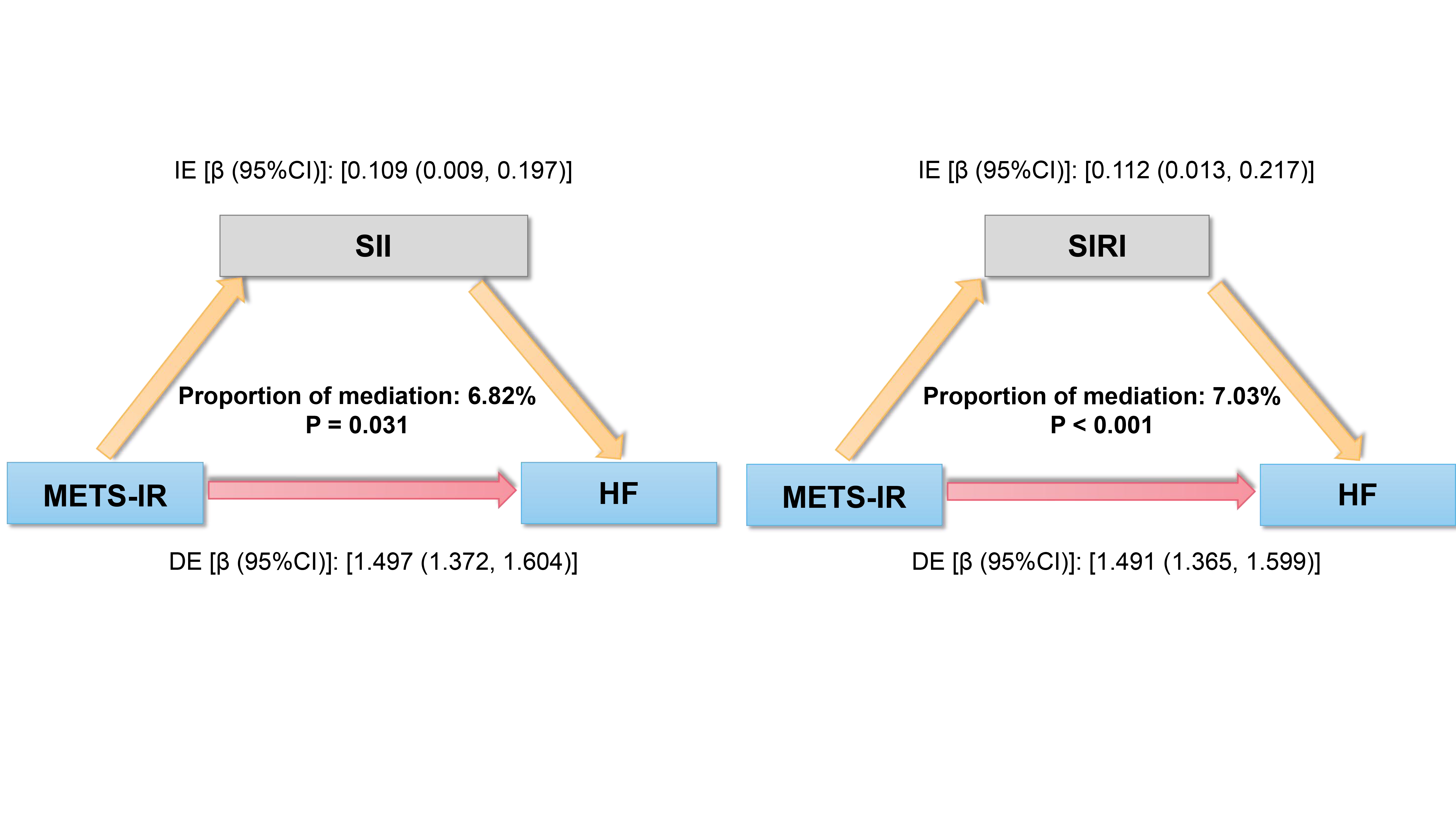


**Figure S3.** Systemic inflammatory indicators in mediation
